# Manipulation of Cannabinoid Biosynthesis *via* Transient RNAi Expression

**DOI:** 10.3389/fpls.2021.773474

**Published:** 2021-12-10

**Authors:** Lennon Matchett-Oates, German C. Spangenberg, Noel O. I. Cogan

**Affiliations:** ^1^Agriculture Victoria, AgriBio, The Centre for AgriBioscience, Bundoora, VIC, Australia; ^2^School of Applied Systems Biology, La Trobe University, Bundoora, VIC, Australia

**Keywords:** *Cannabis sativa*, RNAi, cannabinoid biosynthesis genes, *agrobacterium*, post-transcriptional gene silencing, THCAS, CBDAS, CBCAS

## Abstract

*Cannabis sativa* L. produces unique phytocannabinoids, which are used for their pharmaceutical benefits. To date, there are no reports of *in vivo* engineering targeting the cannabinoid biosynthesis genes to greater elucidate the role each of these genes play in synthesis of these medically important compounds. Reported here is the first modulation of cannabinoid biosynthesis genes using RNAi *via* agroinfiltration. Vacuum infiltrated leaf segments of the Cannbio-2 *C. sativa* strain, transfected with different RNAi constructs corresponding to *THCAS, CBDAS*, and *CBCAS* gene sequences, showed significant downregulation of all cannabinoid biosynthesis genes using real-time quantitative PCR. Using RNAi, significant off-targeting occurs resulting in the downregulation of highly homologous transcripts. Significant (*p* < 0.05) downregulation was observed for *THCAS* (92%), *CBDAS* (97%), and *CBCAS* (70%) using pRNAi-GG-*CBDAS-UNIVERSAL*. Significant (*p* < 0.05) upregulation of *CBCAS* (76%) and non-significant upregulation of *THCAS* (13%) were observed when transfected with pRNAi-GG-*CBCAS*, suggesting the related gene’s ability to synthesize multiple cannabinoids. Using this approach, increased understanding of the relationship between cannabinoid biosynthesis genes can be further elucidated. This RNAi approach enables functional genomics screens for further reverse genetic studies as well as the development of designer cannabis strains with over-expression and/or downregulation of targeted cannabinoid biosynthesis genes. Functional genomics screens, such as these, will further provide insights into gene regulation of cannabinoid biosynthesis in *Cannabis*.

## Introduction

*Cannabis sativa* L. is one of the earliest domesticated and cultivated plants with records of its use in central Asia dating back more than 6,000 years (Li, 1973). Cannabis belongs to the *Cannabaceae* family and has been used for millennia for its source of bast fiber, seed oil, food, and psychoactive constituents for recreational and medicinal purposes (Touw, 1981). Cannabis produces more than 120 cannabinoids, which are unique secondary metabolites found only in cannabis (ElSohly et al., 2017). Cannabis contains a unique Cannabinoid biosynthesis pathway which produces biologically inactive compounds, such as Tetrahydrocannabinolic acid (THCA) and Cannabidiolic acid (CBDA; Matchett-Oates et al., 2021a) which when decarboxylated are converted to their biologically active forms Δ^9^-tetrahydrocannabinol (THC) and Cannabidiol (CBD) displaying psychoactive and non-psychoactive properties, respectively (Kogel et al., 2018). Other major cannabinoids of interest produced are cannabigerol (CBG), cannabichromene (CBC), cannabinol (CBN), and tetrahydrocannabivarin (THCV). The pharmacological effects of these cannabinoids have been of great interest due to the affinity these chemical compounds have for the endogenous cannabinoid system receptors (Movahedi et al., 2015). The use of medicinal cannabis in the treatment of conditions, including pain management (Campbell et al., 2001), cancer (Machado Rocha et al., 2008), multiple sclerosis (Rog et al., 2005), and epilepsy (Russo, 2017), has been widely reviewed. THC has been the primary cannabinoid studied in cannabis research since its discovery (Gaoni and Mechoulam, 1964), but now considerable interest exists in understanding the activity of the other major cannabinoids and their possible therapeutic properties. More specifically, the common precursor of all cannabinoids is CBG, which is enzymatically synthesized into the unique phytocannabinoids, giving cannabis its therapeutic potential (Borrelli et al., 2013).

The dioecious, wind pollination nature of cannabis has created a highly diverse genetic pool in which strains are generated in clandestine breeding efforts, creating a highly diverse population with high levels of sequence and copy number variations affecting the drug content (Weiblen et al., 2015; Matchett-Oates et al., 2021a). Cannabis can be classified into different chemotypes according to their CBD:THC ratio (Pacifico et al., 2006). THCA synthase (THCAS) and CBDA synthase (CBDAS) are the competing enzymes for the common precursor, cannabigerolic acid (CBGA), which determines the chemotype of cannabis plants. The loci containing these synthase genes have recently been resolved showing that as many as 13 synthase gene copies reside within chromosome 7 (Grassa et al., 2018). Further comparison of publicly available cannabis genomes shows that there is significant variation in total synthase gene copy number with sequence homology between all genes being greater than 90% (Grassa et al., 2021; Matchett-Oates et al., 2021a). It is this variation and tightly linked regions that makes the cannabinoid biosynthesis pathway complex to engineer with the intent to create novel designed chemotypes of cannabis for therapeutic uses. Such examples to engineer the cannabinoid pathway within yeast to produce cannabinoids are already possible (Luo et al., 2019), though the adaptation of this approach toward medical applications is still yet to be addressed.

Development of new cannabis strains for medicinal purposes through traditional breeding efforts is a lengthy and expensive process. The use of targeted gene silencing tools to accurately and efficiently knockdown targeted gene expression will enable the generation of novel cannabinoid profiles. The development of genetically modified plants raises public concern for their potential consequences on human health. An alternative when using RNAi is the application of exogeneous dsRNA to induce gene silencing without risking societal acceptance. However, the majority of studies regarding exogenous application of dsRNA is rarely applied under open-field conditions assessing the environmental factors affecting RNAi efficacy, with such practices currently unperformed using cannabis. The use of RNAi is not considered genetically modified through some regulatory agencies (Office of the Gene Technology Regulator, 2018), which can improve the end point consumers opinions regarding novel chemotypes developed using RNAi technologies. Through genome-wide association studies on *THCAS* and *CBDAS* loci, it has been shown that a cannabis variety with a functional *THCAS* but a non-functional *CBDAS* locus is possible (Welling et al., 2020). Conversely, a cannabis variety with a non-functional *THCAS* locus has not been discovered, indicating trace levels of THC will always be produced, such is the case with hemp. Using gene silencing tools, designer strains with high levels of CBD producing zero THC are possible, as are strains with elevated levels of CBG, which contains anti-cancer properties (Borrelli et al., 2014), through the knockdown of the downstream enzymatic processes of *THCAS, CBDAS*, and *CBCAS*. The use of environmental pressures applied through varying nutrient concentrations (Saloner and Bernstein, 2021; Shiponi and Bernstein, 2021) or light spectrum and lighting source (Magagnini et al., 2018; Namdar et al., 2019) has previously demonstrated significant modulation of secondary metabolites, up to 300% in some instances (Shiponi and Bernstein, 2021). While this ability to variably control cannabinoid content in cannabis using environmental conditions is significant, the synergistic effects of all cannabinoids either increasing or decreasing make this approach incapable of producing a complete knockdown/significant downregulation of specific cannabinoids to create novel chemotypes. The generation of stably transformed lines is a lengthy process, requiring protocol development for transformation and regeneration. Transient expression systems are widely used as a valuable tool for vector construct evaluation, all the while being fast and inexpensive with specific protocols in cannabis already developed (Schachtsiek et al., 2019; Deguchi et al., 2020) exploring dsRNA and virus-induced gene silencing mechanisms, with significantly downregulated targeted gene expression levels observed. RNAi transient gene suppression is a well-characterized method for reverse genetics and can allow for rapid screening of RNAi constructs for later stable transformation using *Agrobacterium*. Intron-containing hairpin RNA (ihpRNA) are used to induce degradation of targeted genes using RNAi mechanisms. The generation of small interfering RNA (siRNA), from dsRNA by Dicer-like proteins (DCLs), binds to the RNA-induced silencing complex (RISC), with one strand of the siRNA acting as a guide, targeting mRNA which share a complementary sequence (Majumdar et al., 2017). Once base pairing occurs, Argonaute (AGO) proteins cleave the target mRNA thus preventing transcription translation. This RNAi mechanism was first shown to be highly effective (Waterhouse et al., 1998) and has since been widely used for silencing endogenous and viral RNA in many plant species (Younis et al., 2014).

Limited reports of transient expression systems in cannabis exist. Recently, GFP has been transiently expressed in mesophyll protoplasts of cannabis with over 20% transformation efficiency (Matchett-Oates et al., 2021b). *Agrobacterium*-mediated transformation protocols have previously been used for the stable transformation of hairy roots cultures to express *β*-glucuronidase (GUS; Wahby et al., 2013) and expression of phosphomannose isomerase (PMI) in friable callus (Feeney and Punja, 2003). More recently, transient RNAi *Agrobacterium*-mediated transformation of cannabis has been reported (Schachtsiek et al., 2019). Virus-induced gene silencing, utilizing *Cotton leaf crumple virus* (CLCrV), showed transcriptional silencing in virus affecting genes. Optimization of variables involved in transient *Agrobacterium*-mediated transformation has also been explored using heterologous expression of GUS and GFP in multiple tissue types (Deguchi et al., 2020). To our knowledge, this article is the first to report the use of transient expression RNAi constructs in cannabis to silence the medically important cannabinoid biosynthesis genes. The interaction between the highly homologous genes and the ability to silence all related genes using a single construct is also described. Successful silencing of the conserved homologous biosynthesis genes enables us to unravel gene function and their relationships within this important biosynthetic pathway.

## Materials and Methods

### Plant Material and Growth Conditions

All research was performed under Medicinal Cannabis Research Licence (RL011/18) and Permit (RL01118P4) issued through the Department of Health (DoH), Office of Drug Control (ODC) Australia.

Leaf material from the *C. sativa* cultivar “Cannbio-2” (1,1.8, THC,CBD) was used for transient expression experiments. Cannbio-2 plants were propagated in 9-L plastic pots using coco-coir and grown using hydroponics nutrients coco A+B (THC^®^, Australia) as per manufacturer’s recommended nutrient strength, in a controlled greenhouse environment at 25°C day time temperature, 20°C night time temperature, 50-60% humidity. Leaf explants were chosen from young, newly developing shoot apical meristems from the top half of the plant. Leaf explants were chosen from young, newly developing shoot apical meristems from the top half of the plant on approximately 2-month-old donor plants grown under high pressure sodium grow lights (Papillon, Holland), 500 μmol m^−2^ s^−1^, with a photoperiod of 18-h light and 8-h dark regime.

### Identification of Candidate Genes, siRNA Design, and Gene Amplification

Sequence data of the endogenous *THCAS*, *CBDAS*, and *CBCAS* genes were accessed from the Cannbio-2 genome assembly (Braich et al., 2020; https://doi.org/10.46471/gigabyte.10; BioProject: PRJNA667278). *THCAS*, *CBDAS*, and *CBCAS* gene sequences were determined by BLAST querying the Cannbio-2 genome assembly with an e-value threshold set at <10^−10^. Exons from the gene sequences were predicted using FGENESH (Solovyev et al., 2006) and ExPASy (Gasteiger et al., 2003). Predicted gene sequences were viewed and aligned using Geneious Prime 2020.2.^1^ siRNAs from amplified gene sequences were predicted using pssRNAit,^2^ using the software’s recommended parameters, to generate a library of siRNA fragments within the chosen gene sequences (Supplementary Data). The number of predicted off-target sites within the Cannbio-2 cannabinoid biosynthesis genes was performed by BLASTn analysis of each siRNA sequence, recording the total number of exact sequence homology matches, with off-targeting determined as an exact sequence residing within a different biosynthesis gene set. In the instance of pRNAi-GG-*CBDAS-UNIVERSAL* an off-target is defined as an exact match that does not reside within the CBDAS-truncated#4 homolog.

Primers were designed, using Primer3 (Untergasser et al., 2012), in gene regions of sequence variance and homology, with products between ~250 and ~600 base pairs for siRNA generation *in vivo* (Supplementary Data). Each forward and reverse primer had the 5' adapter sequences “acca ggtctc aggag” and “acca ggtctc atcgt,” respectively. DNA fragments were PCR-amplified from Cannbio-2 genomic DNA, using Phusion polymerase (New England Biolabs, Ipswich, MA) with PCR cycling as follows: 98°C 30 s, 35 cycles of 98°C 10 s, 60°C 30 s, 72°C 30 s, and final extension 72°C 10 min.

### Plasmid Construction, *Agrobacterium* Culture Conditions, and Vacuum Infiltration

For expression of siRNAs, pRNAi-GG vector was used within this study. pRNAi-GG was provided by The Arabidopsis Biological Resource Center (TAIR). The construction of the vectors containing gene sequences of interest was followed according to a previously published protocol (Yan et al., 2012). Briefly, 50 ng of purified PCR products was mixed with 200 ng of pRNAi-GG with 5 units of Bsal (New England Biolabs, Ipswich, MA) and 10 units of T4 Ligase (New England Biolabs, Ipswich, MA) in a total volume of 20 μl in T4 ligation buffer. Restriction-ligation was carried out at 37°C for 2 h followed by a final digestion at 50°C for 5 min and heat inactivation at 80°C for 5 min. *E. coli* DH5α competent cells were transformed with 5 μl of the mixture and plated on LB media containing 25 mg/L kanamycin and 5 mg/L chloramphenicol.

Recombinant bacterial colonies were PCR verified with primers flanking the PCR product insert, and bands were visualized using a TapeStation 2200 (Agilent, Santa Clara, CA) with colonies of expected band sizes sequence verified. Final constructs were labelled pRNAi-GG-*THCAS*, pRNAi-GG-*CBDAS*, pRNAi-GG-*CBCAS*, and pRNAi-GG-*CBDAS-UNIVERSAL* (Supplementary Data).

Recombinant *Agrobacterium tumefaciens* strains were generated *via* electroporation following a previously published protocol (Lin, 1995). *Agrobacterium* culture conditions and vacuum infiltration protocols were performed using a previously reported protocol (Deguchi et al., 2020) with slight modifications. In summary, for the expression of pRNAi-GG constructs, *A. tumefaciens* strain GV3101 was used for transient expression experiments. Recombinant *A. tumefaciens* were inoculated and grown in YM media (0.5 g/L K_2_HPO_4_, 0.2 g/L MgSO_4_-7H_2_O, 0.1 g/L NaCl, 10 g/L mannitol, 0.4 g/L yeast extract, PH 7; Sigma-Aldrich, St. Louis, MO) overnight at 220 rpm at 30°C. The culture was centrifuged at 4,000 *g* for 10 min and resuspended to an OD_600_ = 0.5 in infiltration media (10 mM MES, 1x MS and vitamins, 2% glucose, 200 μM acetosyringone, pH 5.6; Sigma-Aldrich, St. Louis, MO) and placed on a rotary shaker (Ratek, Australia) for 2 h prior to vacuum infiltration. Immediately before infiltration, 5 mM ascorbic acid, 0.05% (v/v) Pluronic F-68, and 0.015% (v/v) Silwet L-77 (Sigma-Aldrich, St. Louis, MO) was added to the *A. tumefaciens* culture.

Leaf segments (approx. 2 cm × 2 cm) were taken from young fully expanded leaves of *ca*. 2-month-old, donor Cannbio-2 plants and placed in a Petri dish (100 mm × 15 mm) containing *A. tumefaciens* suspension. The Petri dish was then placed in a desiccator (Tarsons, West Bengal, India) for 2 min at 400 mbar with vacuum pressure gently released. Vacuum was reapplied once more allowing thorough infiltration. Leaf material was washed with sterile water and transferred onto moist (ddH_2_O) filter paper (Whatman, Maidstone, United Kingdom) in a Petri dish and placed in a controlled environment room at 24°C with an 18 h photoperiod for 4 days.

### Quantitative Real-Time PCR Analysis of Agroinfiltrated Leaf Segments

Seventy-two hours post-vacuum agroinfiltration, leaf segments were snap frozen in liquid nitrogen and total RNA was extracted following manufacturer’s instructions (RNeasy Plant Mini Kit, Qiagen, Hilden, Germany). cDNA synthesis and qPCR were carried out in one step with Luna Universal One-Step RT-qPCR Kit (New England Biolabs, Ipswich, MA) following manufacturer’s instructions. Quantitative PCR parameters used were as follows: 95°C for 60 s, 40 cycles at 95°C for 15 s, and 59°C for 15 s carried out with a CFX-96 Touch Real-Time PCR Detection System (Bio-Rad, Hercules, CA). Melting curves were measured, and gene expression levels were calculated from the cycle threshold according to the 2^−ΔΔCt^ method (Livak and Schmittgen, 2001). Paired t test was performed (*p* = 0.05) to determine significance using RStudio (version 1.1.453, RStudio Inc., Boston, MA). The *UBQ5* gene was used as an internal reference (Deguchi et al., 2020), with three biological replicates used for all qPCR experiments with two technical replicates. All primer sequences are listed in Supplementary Data.

## Results

### Identification of Cannabinoid Genes and siRNAs Prediction

**Figure 1:**
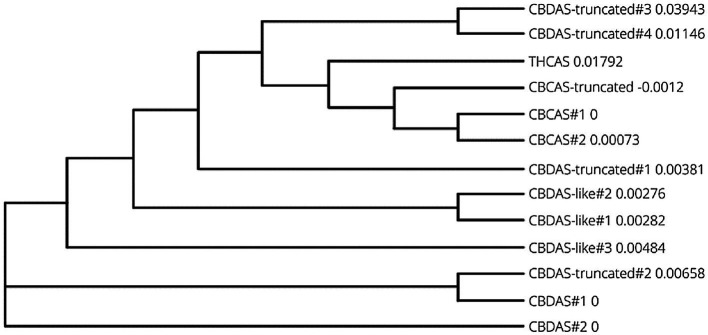
Phylogenetic tree of coding sequence data from cannabinoid biosynthesis genes in Cannbio-2 displaying highly homologous nature of gene homologs.

To establish RNAi in *C. sativa*, *THCAS*, *CBDAS*, and *CBCAS* gene sequences were determined by BLAST querying the Cannbio-2 genome sequence assembly with publicly available sequences (Table 1). Each cannabinoid biosynthesis gene, and accompanying homologs, were analyzed for functionality using FGENESH and ExPASy and subsequently BLASTn analyzed for homology to publicly available sequences and pairwise aligned using MUSCLE to create a phylogenetic tree (Figure 1) and a matrix with identity percentages of coding sequences (Table 2).

**Table 1:** Cannbio-2 analysis of cannabinoid biosynthesis genes with PCR amplification, copy number, and siRNA prediction information.

Cannabinoid biosynthesis gene	Accession number/Source of query	Copy number/homologs	Primer pairs used for amplification	Product size	Predicted siRNA #
THCAS	AB057805	1	F: AACTATTTTATGCTCTAAGAAAGT R: TTTGTTATGAAGTGAGTCATGA	603 bp	93
CBDAS	AB292682	9	F: AAGTCCCATTTGTTATAGTAGA R: TTGACAAGCTCATGTATCTC	442 bp	70
CBCAS	Publication number: WO/2015/196275	3	F: GGCCAGTATTCTCTGCTC R: CTAGTTCTGAAGTGAGTCGTG	606 bp	95
CBDAS-UNIVERSAL	-	-	F: CCGGAGCTACCCTT R: GGCTATACGTGGTGG	247 bp	38

**Table 2:** Identity matrix of cannabinoid biosynthesis genes coding sequences in Cannbio-2 global alignment.

	THCAS												
THCAS		CBCAS#1											
CBCAS#1	96.31		CBCAS#2										
CBCAS#2	96.23	99.93		CBCAS-truncated									
CBCAS-truncated	95.88	99.90	99.79		CBDAS-like#1								
CBDAS-like#1	93.10	92.38	92.29	91.83		CBDAS-like#2							
CBDAS-like#2	93.10	92.38	92.29	91.83	100.00		CBDAS-like#3						
CBDAS-like#3	92.98	92.28	92.20	91.71	99.51	99.51		CBDAS-like#4					
CBDAS-like#4	92.60	92.03	91.96	91.44	99.51	99.51	99.44		CBDAS-like#5				
CBDAS-like#5	92.37	91.64	91.56	91.03	99.49	99.49	99.26	99.11		CBDAS-truncated#1			
CBDAS-truncated#1	92.19	92.74	92.74	52.19	98.96	98.96	98.44	98.96	98.54		CBDAS-truncated#2		
CBDAS-truncated#2	72.86	71.35	71.28	61.84	74.38	74.38	74.80	76.62	75.72	98.44		CBDAS-truncated#3	
CBDAS-truncated#3	86.79	86.66	86.66	86.42	87.01	87.01	86.90	86.90	86.87	84.74	56.28		CBDAS-truncated#4
CBDAS-truncated#4	67.78	66.70	66.60	62.03	66.14	66.14	67.17	67.59	67.39	90.00	82.72	57.83	

Within the Cannbio-2 genome, a single functional copy of *THCAS* exists; however, *CBDAS* and *CBCAS* contain nine and three homologs/pseudogenes, respectively. Using FGENESH and ExPASy, two identical, full-length potentially functional *CBDAS* cannabinoid biosynthesis genes were discovered (CBDAS-like#1 and #2), and three homologs were identified containing several single nucleotide polymorphisms (SNPs) leading to differences in predicted protein translations (CBDAS-like#3-5), however full length and potentially functional, and four copies of *CBDAS* were found to be truncated when proteins were predicted (CBDAS-truncated#1-4). The coding sequences (CDS) of each *CBDAS* homologs were aligned, and non-truncated homologs are shown to be >86% homologous. The high levels of sequence similarity of the *CBDAS* homologs (Table 2) at the DNA level, and regardless of the size of the PCR insert for siRNA generation, sequence homology is too significant to identify one best-fit homolog for vector design, and thus, a single homolog of *CBDAS* was chosen, identified as CBDAS-like#1 within the Cannbio-2 genome (Supplementary Data), for pRNAi-GG-*CBDAS* vector construction.

**Figure 2:**
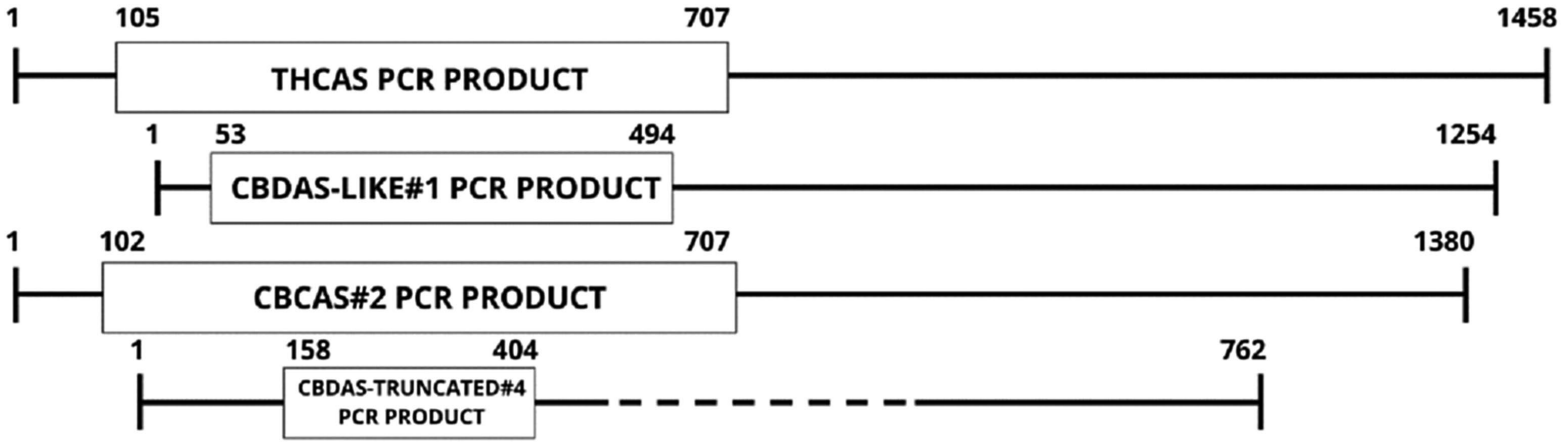
Graphical representation of gene CDS alignments used for PCR amplification for siRNA generation.

Two full-length, potentially functional copies of *CBCAS* were found (CBCAS-like#1 and #2) having identical sequence homology, except for base pair 482, where a synonymous SNP occurs (T to C); however, this does not affect predicted translated proteins (Supplementary Data). A truncated *CBCAS* homolog was also discovered at only 969 bp designated CBCAS-truncated. CBCAS#2 was chosen within the Cannbio-2 genome for pRNAi-GG-*CBCAS* vector construction (Supplementary Data). A significantly smaller sequence (247 bp; Supplementary Data), homologous to the CBDAS-truncated#4 homolog, was chosen in a region of high homology from the sequence alignment of all cannabinoid synthesis genes CDS, however lower in homology (<90%) within the subset of *CBDAS* sequences, designated “*CBDAS-UNIVERSAL”* to determine whether a smaller gene sequence for RNAi containing lower homology could be more effective in gene silencing through off-targeting. A graphic representation for the alignment of cannabinoid biosynthesis genes, with the PCR products sizes, is shown in Figure 2.

The gene sequences selected for RNAi were analyzed using pssRNAit to assess the degree of off-targeting to the identified cannabinoid gene sequences for each specific vector. Efficient gene silencing requires the formed siRNA to contain minimal off-targeting silencing effects. From the amplified *THCAS* sequence, 93 siRNA were predicted with 1,609 potential off-targets, *CBDAS* with 70 predicted siRNA and 1,609 potential off-targets, *CBCAS* with 95 predicted siRNA and 1,647 potential off-targets, and *CBDAS-UNIVERSAL* with 38 predicted siRNA with 630 potential off-targets (Table 1).

To filter out irrelevant off-target sites not residing within the cannabinoid genes, each siRNA was aligned to Cannbio-2 cannabinoid biosynthesis genes for sequence similarity to greater understand off-targeting potential within these highly homologous sequences. A total number of 369 exact targets for pRNAi-GG-*THCAS* exist within Cannbio-2 cannabinoid biosynthesis genes with 93 exact matches to *THCAS* and 276 off-targets existing within the other gene sets (Table 3). pRNAi-GG-*CBDAS* contained 447 total exact targets within all biosynthesis genes, with 381 targeting a minimum of 1 *CBDAS* homologs and containing considerably more off-targets tallying 64 sites not residing within *CBDAS* homologs (Table 3). pRNAi-GG-*CBCAS* contained a similar number of total targets, 428, with 276 targets within CBCAS homologs and contained substantially more off-targets, with 152 exact matches across other gene sets (Table 3). Within the pRNAi-GG-*CBDAS-UNIVERSAL* predicted siRNA, only 69 exact targets exist within all biosynthesis genes. A total of 38 siRNA sites exist within the predicted CBDAS-truncated#4 gene sequence, with the remaining 31 target sites residing within *CBDAS* homologs (Table 3).

**Table 3:** Off-targeting frequency in each cannabinoid gene from generated siRNA in each vector.

RNAi vector	Cannabinoid biosynthesis genes												
	THCAS	CBCAS#1	CBCAS#2	CBCAS-truncated	CBDAS-like#1	CBDAS-like#2	CBDAS-like#3	CBDAS-like#4	CBDAS-like#5	CBDAS-truncated#1	CBDAS-truncated#2	CBDAS-truncated#3	CBDAS-truncated#4
pRNAi-GG-*THCAS*	93	53	53	51	18	18	16	23	17	0	19	0	6
pRNAi-GG-*CBDAS*	18	16	16	14	64	64	70	54	54	0	60	0	15
pRNAi-GG-*CBCAS*	53	95	95	86	16	16	16	18	12	0	16	0	5
pRNAi-GG-*CBDAS-UNIVERSAL*	0	0	0	0	5	5	5	5	5	0	5	1	38

### Vector Construction, Generation of Recombinant *Agrobacterium*, and Vacuum Infiltration

To test the efficiency of silencing cannabinoid biosynthesis genes, recombinant expression vectors were made for the four target sequences. The vectors contained sense-antisense orientation separated by an intron and were cloned into an *E. coli* strain.

Eight recombinant colonies were chosen, for each treatment, for colony PCR using sequence-specific primers residing within the specific sequence and residing on the vector backbone. All clones showed the expected bands confirming the correct inserts, which were subsequently sequenced to confirm the correct sequences as expected.

*Agrobacterium* strain, GV3101, was chosen for *Agrobacterium*-mediated transient expression in leaf segments of Cannbio-2. Recombinant pRNAi-GG vectors were transformed into GV3101 with appropriate selection. Agroinfiltration was achieved using vacuum infiltration on the excised cannabis leaf segments optimized for use with Cannbio-2 leaf material.

### Silencing of Cannabinoid Biosynthesis Genes

Leaf segments of *C. sativa* Cannbio-2 strain were infiltrated with recombinant *A. tumefaciens* and incubated in a climate-controlled environment. To investigate the extent of downregulation of the cannabinoid biosynthesis genes, quantification of the transcript levels of *THCAS*, *CBDAS*, and *CBCAS* was performed using qPCR. Each genes expression level was analyzed in three biological replicates and two technical replicates with gene primer pairs located upstream of the respective RNAi construct design.

Using the reference gene *UBQ5* for normalization in all qPCR experiments, infiltrated leaf segments saw varying levels of downregulation in all cannabinoid biosynthesis genes, and in one instance, upregulation of *THCAS* and *CBCAS* in response to RNAi transient expression compared to leaf segments infiltrated with disarmed *Agrobacterium* as negative controls.

**Figure 3:**
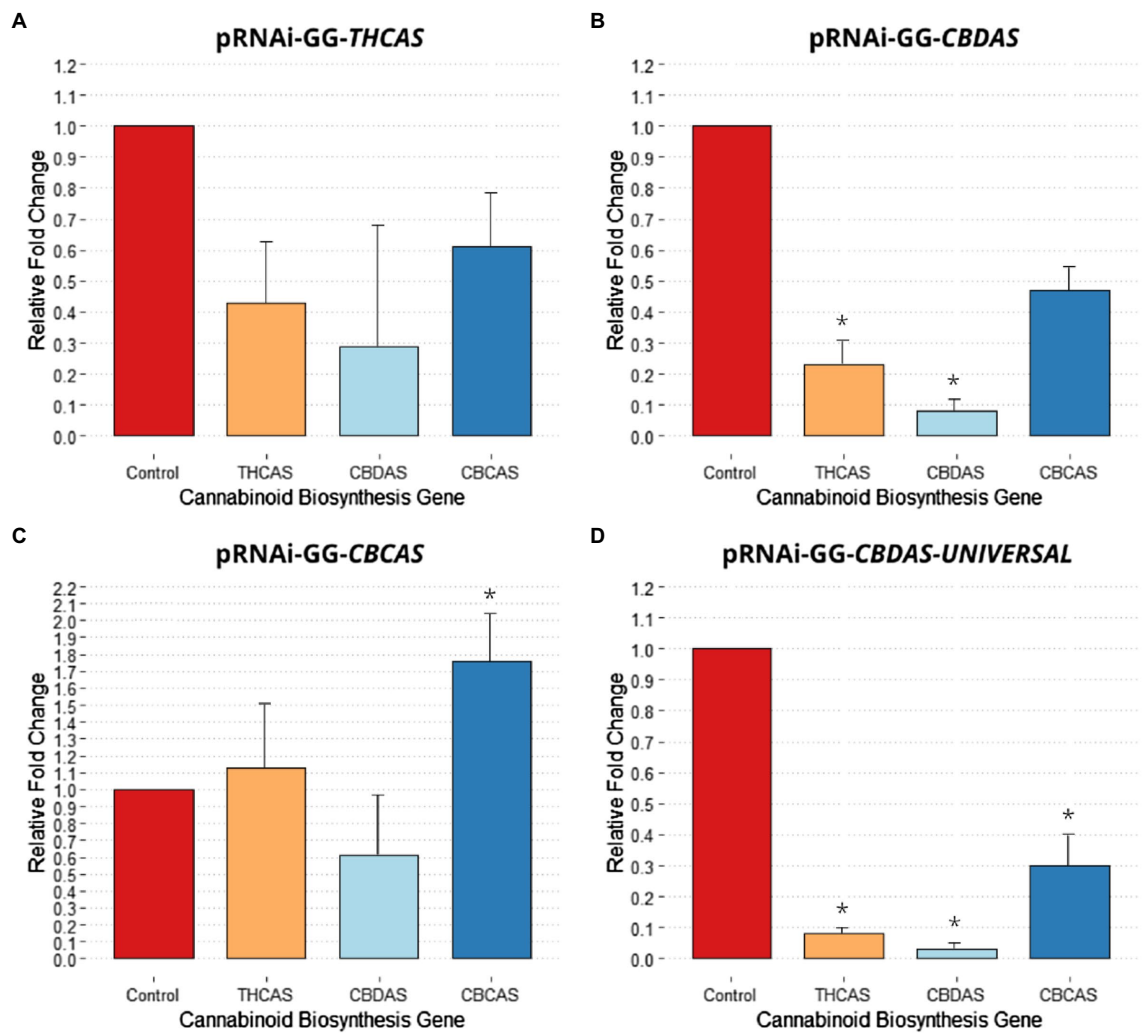
Effect of different pRNAi-GG vectors on cannabinoid biosynthesis gene relative expression change. **(A)** Relative fold change post agroinfiltration with pRNAi-GG-*THCAS*. **(B)** Relative fold change post agroinfiltration with pRNAi-GG-*CBDAS*. **(C)** Relative fold change post agroinfiltration with pRNAi-GG-*CBCAS*. **(D)** Relative fold change post agroinfiltration with pRNAi-GG-*CBDAS*-*UNIVERSAL*. Significance is determined by paired *t*-test, (*p* < 0.05) is denoted by *. Error bars represented SE.

Agroinfiltration with pRNAi-GG-*THCAS* successfully downregulated *THCAS*, *CBDAS*, and *CBCAS*. From the qPCR data, pRNAi-GG-*THCAS* saw a 57% reduction in *THCAS* transcript levels (Figure 3A). Interestingly, using the *THCAS* gene sequence for RNAi, between the vectors, was ranked the 3rd most effective for downregulating the targeted gene. Off-targeting of this vector construct caused downregulation of *CBDAS* with a 71% reduction (non-significant, *p* = 0.48) in transcript levels making this, also, the 3rd most effective in downregulating *CBDAS*. The highly homologous sequence of *CBCAS* saw a more conserved reduction of 39% (non-significant, *p* = 0.45) in transcript levels, with the off-targeting effect of this vector ranking it also third in silencing *CBCAS*.

Agroinfiltration with pRNAi-GG-*CBDAS* downregulated the three cannabinoid biosynthesis genes more effectively, comparatively. The pRNAi-GG-*CBDAS* vector saw a significant (*p* < 0.05) reduction of CBDAS with 92% downregulation (Figure 3B), making it the second most effective vector for downregulation of *CBDAS* behind pRNAi-GG-*CBDAS-UNIVERSAL*. Significant (*p* < 0.05) off-target downregulation of *THCAS* was observed with a 77% reduction in transcript levels, making this more efficient in inadvertent downregulation of *THCAS* than using the gene-specific sequence of *THCAS* to produce siRNA. Increased downregulation of *CBCAS* was also observed, with a 53% reduction (non-significant, *p* = 0.07) in transcript levels compared to the control, making this vector the second most effective construct for silencing *CBCAS*.

Agroinfiltration with pRNAi-GG-*CBCAS* was least effective in silencing cannabinoid biosynthesis genes, which conversely saw non-significant upregulation of *THCAS* and *CBCAS* transcript levels (Figure 3C). pRNAi-GG-*CBCAS* saw a 39% downregulation (non-significant, *p* = 0.22) of *CBDAS*, making it the least effective vector for *CBDAS* silencing. Interestingly, off-targeting caused *THCAS* to be upregulated by 13% (non-significant, *p* = 0.42) compared to the control regardless of the >96% homology shared between the two genomic sequences. This increase makes this the least effective vector for *THCAS* silencing. *CBCAS* transcript levels were significantly (*p* < 0.05) upregulated 76% using the targets gene sequence, rendering it least effective of all vectors for gene silencing of *CBCAS*.

Agroinfiltration with pRNAi-GG-*CBDAS-UNIVERSAL* was significantly more efficient in downregulating *THCAS*, *CBDAS*, and *CBCAS*. The small construct, homologous to a highly conserved region of the aligned gene sequences, saw comparatively dramatic decreases of transcript levels compared to the other constructs (Figure 3D). A significant (*p* < 0.05) downregulation of *THCAS*, with a 92% reduction in transcript levels, was observed due to off-targeting, making this vector highly effective in targeting *THCAS*. A significant (*p* < 0.05) reduction in *CBDAS* was also observed, with a 97% reduction in transcript levels compared to the control. Like pRNAi-GG-*CBDAS*, pRNAi-GG-*CBDAS-UNIVERSAL* is most effective in silencing the targeted gene used to create the vector construct (CBDAS-truncated#4), also making this smaller construct the most effective in downregulating *CBDAS*. Significant (*p* < 0.05) reduction in *CBCAS* was also observed, with a 70% decrease in transcript levels compared to the control. This off-targeting effect makes this vector the most effective in silencing *CBCAS* compared to the other vectors.

## Discussion

Genetic transformation of cannabis has only recently been achieved using *Agrobacterium* (Schachtsiek et al., 2019; Deguchi et al., 2020). Induced RNA silencing by hairpin-loop RNAi constructs have previously been optimized through the exploration of variables involved in vacuum infiltration by measuring relative GUS expression (Deguchi et al., 2020). Building upon the approach developed by Deguchi et al. (2020), vacuum infiltration was achieved in leaf segments of Cannbio-2, a cultivar with a ratio 1:1.8 THC to CBD, to significantly reduce the relative expression of cannabinoid biosynthesis genes *THCAS*, *CBDAS*, and *CBCAS*. This work is the first successful downregulation of these cannabinoid biosynthetic genes, showing that the use of RNAi constructs with the gene sequences of each gene, respectfully, results in varying levels of suppression.

In this paper, the downregulation of cannabinoid biosynthesis genes was evaluated using vacuum agroinfiltration. Using the common Golden Gate Cloning method to construct RNAi vectors, with sense and antisense sequence inserts, downregulation of *THCAS*, *CBDAS*, and *CBCAS* was observed to varying effectiveness. In this study, it was hypothesized that using large (400–600 bp) RNAi constructs to silence-specific cannabinoid biosynthesis genes would result in a downregulation of the other highly homologous gene sequences due to siRNA off-targeting. Observing the relative transient expression levels of the targeted genes 4 days post-agroinfiltration with pRNAi-GG-*THCAS* saw a downregulation of 57, 71 and 39% of *THCAS*, *CBDAS*, and *CBCAS*, respectfully (Figure 3A). The siRNA generated using pRNA-GG-*THCAS* targeted substantially more regions within *THCAS* and *CBCAS* compared to *CBDAS* (Table 2). While the results were all non-significant due to the variance between treated samples, off-targeting is still prevalent as demonstrated by the ability to downregulate non-specific targets. This confirmation of the hypothesis can be explained by the highly homologous (>90%) gene sequences, which when amplified and used in RNAi, will produce siRNA (Table 1) that will have significant off-targeting. siRNA predicted from the amplified *THCAS* sequence were more effective in downregulating the *CBDAS* transcripts, comparatively, to *THCAS* and *CBCAS*, which are more highly sequence homologous (>96%) than *CBDAS* is to *THCAS* (92%). The most likely explanation for this increased downregulation of *CBDAS* would be the fact that Cannbio-2 contains 5 potentially functional copies. Within the Cannbio-2 genome (Braich et al., 2020), a fully functional *CBDAS* gene is absent due to assembly error within the retrotransposon regions in a hybrid genotype. Cannbio-2 does contain an identical *CBDAS* gene within the transcriptome (Braich et al., 2019; Cannbio_016865); however, this is not present within the genome. However, several full-length, potentially functional *CBDAS* homologs exist in which their function is yet to be determined. The increased copy number of *CBDAS* is due to the cannabinoid biosynthesis genes being arranged in tandem arrays in long terminal repeat retrotransposons on chromosome 7 (Grassa et al., 2021). The flanking long terminal repeats for *CBDAS* provide an explanation for the movement of the synthase cassette and possible illegitimate recombination resulting in increased synthase numbers. This increased copy numbers will greatly affect RNAi specificity and will result in a higher number of off-targeting sites.

pRNAi-GG-*CBDAS* agroinfiltration qPCR data show significant (*p* < 0.05) downregulation in *CBDAS*, with a reduction of 92% (Figure 3B). Increased downregulation, compared to pRNAi-GG-*THCAS*, was also observed for *THCAS* and *CBCAS*, with 77% (*p* = 0.03) and 53% (*p* = 0.07), respectfully. The presence of 3 *CBCAS* homologs results in a higher number of potential exact targets compared to *THCAS* (Table 3); however, downregulation is twice as effective in *THCAS* than *CBCAS*. Within the genomic sequences and alignment of these two genes and their high level of sequence similarity, it could be expected that the siRNA generated would not contain greater affinity for *THCAS*, but instead downregulate *CBCAS* further due to increased target sites. This, however, is not observed. The increased downregulation despite lower off-target site numbers could be due to the generation of more efficacious siRNAs, which regardless of off-targeting, demonstrate the capability of inhibiting transcription with target sequence variation.

Shorter PCR products for RNAi could also potentially explain higher siRNA efficacy in silencing cannabinoid biosynthesis genes compared to larger inserts. Support of this hypothesis is provided by the qPCR data from agroinfiltration of pRNAi-GG-*CBDAS-UNIVERSAL*, a 247 bp fragment, which produced significant (*p* < 0.05) reduction in *THCAS*, *CBDAS*, and *CBCAS* (Figure 3D). The smaller RNAi construct reduced *THCAS*, *CBDAS*, and *CBCAS* by 92, 97, and 70%, respectfully. Increased efficacy of shorter dsRNA fragments has previously been confirmed in potato (He et al., 2020), with evidence supporting shorter dsRNA length resulting in increased levels of insecticidal protection compared to the larger RNAi constructs investigated. On the contrary, within *Arabidopsis* plants expressing RNAi dsRNA constructs with varying length, there was no observed significant correlation between dsRNA length and reduction of *Fusarium graminearum* infection (Höfle et al., 2020). These studies suggest that within *Cannabis* the effect of dsRNA length and specific region of the gene targeted (e.g., earlier exons) could play a vital role in efficacy, though such assumptions require further investigation and testing.

An additional explanation for the higher efficacy of pRNAi-GG-*CBDAS-UNIVERSAL* is the concentration of more highly effective siRNA, within the shorter sequence, compared to larger fragments which could contain lower efficiency siRNAs. Despite the recent surge in cannabis genome sequencing efforts, the lack of detailed genome sequence annotations and tools to correctly assess the potential for off-targeting of predicted siRNA to the highly homologous cannabinoid biosynthesis gene sequences, as such with the prediction tool “pssRNAit,” requires further investigation. Without the availability of a comprehensive *Cannabis* genome sequence resource to detect the potential off-targeting of these highly homologous genes, the exact sequences of each siRNA were aligned against the Cannbio-2 gene sets and analyzed for off-targeting potential. The limitation of this approach is the inability to correctly evaluate all possible off-targets when slight siRNA sequence variation exists due to the highly homologous nature of all the cannabinoid biosynthesis genes. However, regarding exact siRNA sequence matches residing outside of the intended target, a large number of predicted siRNA produced from pRNAi-GG-*THCAS, CBDAS*, and *CBCAS* exists. Though, interestingly, no exact matches outside of the *CBDAS* homologs are present within any of predicted pRNAi-GG-*CBDAS-UNIVERSAL* siRNA (Table 2). The lower concentration of exact siRNA targets could increase the efficacy of each siRNA, explained by the significant downregulation of *CBDAS*, but it does not explain how this construct is equally capable of significantly downregulating all the highly homologous genes. It is evident that significant off-targeting occurs; however, many base pair differences are tolerated in siRNA targeting is undetermined. Previous work has determined that it is not only the amount of mismatches but also the identity of the matched nucleotides that play an important role in unintended silencing (Du et al., 2005). It was discovered that adenine and cytosine, along with G:U wobble base pair mismatches are silenced with equal efficiency. With these gene sequences being so highly homologous (Figure 1), it is highly probable this would explain the success of pRNAi-GG-*CBDAS-UNIVERSAL*.

Interestingly, contradictory to the proposed hypothesis of collective downregulation of all targeted genes, pRNAi-GG-*CBCAS* agroinfiltration resulted in significant upregulation of *CBCAS* and an observed slight increase in *THCAS*. The 95 predicted siRNAs had a total of 329 exact matches between the CBCAS homologs and THCAS and only 94 matches within the CBDAS homologs resulting increase of 13% in transcript levels of *THCAS* and 76% increase of *CBCAS* transcript levels and a decrease of 39% in *CBDAS* (Figure 3D). An explanation for the upregulation could be the specific sequence containing inefficient siRNA or that the siRNA which did downregulate *CBDAS* triggers a biological response to upregulate the highly similar genes to assist in the enzymatic conversion of CBGA. Alternatively, it is possible that the siRNA generated failed to degrade the mRNA and instead interfered with the translation of THCAS and CBCAS, triggering a feedback loop mechanism leading to increased levels of transcription of these two genes. Examples of such a phenomenon have been observed in mammalian cells (Portnoy et al., 2011; Scacheri et al., 2004) and in wheat lines with RNAi resulting in a compensatory effect increasing total protein content (Gil-Humanes et al., 2008). To date, there are no examples of complete knockdown of individual cannabinoid biosynthesis genes *in vivo* to confirm that specific enzymes can synthesize different cannabinoids. However, multiple cannabinoids have been produced from a single coding sequence of *CBCAS* in yeast through modulating yeast growth conditions (Peet et al., 2016).

Using RNAi to significantly downregulate the medicinally important cannabinoid biosynthesis genes can be achieved using *Agrobacterium*. Much like Deguchi et al. (2020) and Schachtsiek et al. (2019), the use of RNAi in Cannabis to significantly downregulate targeted genes is shown to be possible using different RNAi mechanisms, such as the introduction of dsRNA or virus-induced gene silencing. The drawback from using RNAi to target these genes, and the others previously explored, is the unintended off-targeting, resulting in silencing of the other highly homologous genes. To completely and specifically downregulate a specific enzyme, a sequence-specific genome editing approach, such as CRISPR/Cas-9, would be more applicable by making a large library of constructs and events and then screening for a targeted single gene for knock out (Matchett-Oates et al., 2021a). This approach will allow the investigation into site-specific genome editing events, resulting in a complete knockdown, and whether *in vivo* feedback loops result in gene regulation, through upregulation, in these cannabinoid biosynthesis genes. The use of this agroinfiltration RNAi approach, generating a transformational event resulting in a designer cannabis strain with significantly reduced THC, CBD, and CBC concentrations, is possible. The decreased gene expression will potentially lead to a dramatic increase in the precursor CBGA, which is currently found in minute concentrations, comparatively (Stack et al., 2021). The targeted manipulation of the cannabinoid pathway in this manner could enable the future development of novel genetically modified cannabinoid strains that could deliver new therapeutics pending consumer acceptance of its biotechnology approach. The production of a transgenic cannabis plant using RNAi, in some countries, is not considered genetically modified (Office of the Gene Technology Regulator, 2018), addressing consumer concerns regarding genetic modifications of consumed products.

## Conclusion

Reported within this study is the first downregulation of cannabinoid biosynthesis genes in cannabis using transiently expressed RNAi constructs in leaf segments. This evaluation of RNA silencing efficiency will help further unravel the relationship each cannabinoid biosynthesis gene has through detailed functional genomic screens. This approach can also play an important role in producing stably transformed *C. sativa* designer strains with modulated expression profiles of the medically important cannabinoid biosynthesis genes.

## Data Availability Statement

The original contributions presented in the study are included in the article/Supplementary Material, further inquiries can be directed to the corresponding author.

## Author Contributions

LM-O, NC, and GS provided manuscript conception. LM-O performed data collection and analysis. NC and GS edited the manuscript. All authors contributed to the article and approved the submitted version.

## Funding

This study was funded by Agriculture Victoria Research and Agriculture Victoria Services.

## Conflict of Interest

The authors declare that the research was conducted in the absence of any commercial or financial relationships that could be construed as a potential conflict of interest.

## Publisher’s Note

All claims expressed in this article are solely those of the authors and do not necessarily represent those of their affiliated organizations, or those of the publisher, the editors and the reviewers. Any product that may be evaluated in this article, or claim that may be made by its manufacturer, is not guaranteed or endorsed by the publisher.

## Footnotes

^1^ https://www.geneious.com

^2^ https://plantgrn.noble.org/pssRNAit/
