# Supplementary material for: Manipulation of Cannabinoid Biosynthesis via Transient RNAi Expression
Source: Front Plant Sci. 2021 Dec 10;12:773474. doi: 10.3389/fpls.2021.773474 (PMC8757041; doi:10.3389/fpls.2021.773474)
Supplement: Supplementary file 1 [file Data_Sheet_1.docx]

Supplementary Information

## Cannbio-2 THCAS sequence

## AAAAAATCATTAGGACTGAAGAAAAATGAATTGCTCAGCATTTTCCTTTTGGTTTGTTTGCAAAATAATATTTTTCTTTCTCTCATTCCATATCCAAATTTCAATAGCTAATCCTCGAGAAAACTTCCTTAAATGCTTCTCAAAACATATTCCCAACAATGTAGCAAATCCAAAACTCGTATACACTCAACACGACCAATTGTATATGTCTATCCTGAATTCGACAATACAAAATCTTAGATTCATCTCTGATACAACCCCAAAACCACTCGTTATTGTCACTCCTTCAAATAACTCCCATATCCAAGCAACTATTTTATGCTCTAAGAAAGTTGGCTTGCAGATTCGAACTCGAAGCGGTGGCCATGATGCTGAGGGTATGTCCTACATATCTCAAGTCCCATTTGTTGTAGTAGACTTGAGAAACATGCATTCGATCAAAATAGATGTTCATAGCCAAACTGCGTGGGTTGAAGCCGGAGCTACCCTTGGAGAAGTTTATTATTGGATCAATGAGAAGAATGAGAATCTTAGTTTTCCTGGTGGGTATTGCCCTACTGTTGGCGTAGGTGGACACTTTAGTGGAGGAGGCTATGGAGCATTGATGCGAAATTATGGCCTTGCGGCTGATAATATTATTGATGCACACTTAGTCAATGTTGATGGAAAAGTTCTAGATCGAAAATCCATGGGAGAAGATCTGTTTTGGGCTATACGTGGTGGTGGAGGAGAAAACTTTGGAATCATTGCAGCATGGAAAATCAAACTGGTTGCTGTCCCATCAAAGTCTACTATATTCAGTGTTAAAAAGAACATGGAGATACATGGGCTTGTCAAGTTATTTAACAAATGGCAAAATATTGCTTACAAGTATGACAAAGATTTAGTACTCATGACTCACTTCATAACAAAGAATATTACAGATAATCATGGGAAGAATAAGACTACAGTACATGGTTACTTCTCTTCAATTTTTCATGGTGGAGTGGATAGTCTAGTCGACTTGATGAACAAGAGCTTTCCTGAGTTGGGTATTAAAAAAACTGATTGCAAAGAATTTAGCTGGATTGATACAACCATCTTCTACAGTGGTGTTGTAAATTTTAACACTGCTAATTTTAAAAAGGAAATTTTGCTTGATAGATCAGCTGGGAAGAAGACGGCTTTCTCAATTAAGTTAGACTATGTTAAGAAACCAATTCCAGAAACTGCAATGGTCAAAATTTTGGAAAAATTATATGAAGAAGATGTAGGAGCTGGGATGTATGTGTTGTACCCTTACGGTGGTATAATGGAGGAGATTTCAGAATCAGCAATTCCATTCCCTCATCGAGCTGGAATAATGTATGAACTTTGGTACACTGCTTCCTGGGAGAAGCAAGAAGATAATGAAAAGCATATAAACTGGGTTCGAAGTGTTTATAATTTTACGACTCCTTATGTGTCCCAAAATCCAAGATTGGCGTATCTCAATTATAGGGACCTTGATTTAGGAAAAACTAATCATGCGAGTCCTAATAATTACACACAAGCACGTATTTGGGGTGAAAAGTATTTTGGTAAAAATTTTAACAGGTTAGTTAAGGTGAAAACTAAAGTTGATCCCAATAATTTTTTTAGAAACGAACAAAGTATCCCACCTCTTCCACCGCATCATCATTAATTATCTTTAAATAGATATATTTCCCTTATCAATTAGTTAATCATTATACCATACATACATTTATTGTATATAGTTTATCTACTCATATTATGTATGCTCCCAAGTATGAAAATCTACATTAGAACTGTGTAGACAATCATAAGATATATTTAATAAAATAAATTGTCTTTCTTATTTCAATAGCAAATAAAATAATATTATTTTA

## Cannbio-2 CBDAS-like#1 sequence

## TGATGACGCAGGGGAAGAGGTGGGATGCTTTGTTCGTTTCTAAAGAAATTATCGGGATCAACCTTGGTTTTTACTTTTACTAACCTATTAAAATTTTTACCAAAATACTTTTCACCCCAAATACGTGCTTGTGTGTAATTATTAGGTCTCTCAAAATTAGTTTTTCCTAAATCAAGGTCCCTATAATTGAGATACGCCATTCTTGGATTTTGGGACACATAAGGAGTCGTGAAATTATAAACATTCCGAATCCAGTTTATATGCTTTTCATTATCTTCTTGCTTCTCCCATGAAGCTATGTACCAAATTTCATACATGATTCCAGCTCGATGAGGGAATGGAATTGCTGATTCTGAAATCTCATCCATTATACCACCATAAGGGTAAAACACAAACATCCCAACTCCTACATCTTCTTCATATAATTTTTCCAAAATTGTGACCATTGCGGTTTCTGGAATCGGTTTCTTAACATAGTCTAACTTAATCGAGAAAGCCGCCTTCCGCCCACCTGATCTATCAAGCAAAATTTCTTTTTTAAAATTAGTTGTGTTGTAATTTACAAGACCACTGTAGAAGATGATAGTATCAATCCAGCTCAACTGTTTGCAATCTATTTTTTTAATACCCAATTCAGGAAAGCTCTTGTTCATCAAGTCGACTAGACTATCCACTCCACCATGGAAAATGGAGGAGAAGTAACTGTGTATTGTTGTCTTATTCTTCCCTTGATTATCTGTAATATTCCTGGTTATAAAGTGAGTAAAGAGTAATAATTCTTTTTCATACATGTAAGCAATATTTTGCCATTTGTTAACTAACTTGACAAGCTCATGTATCTCCATGTTCTTTTTAACACTGAATATAGTAGACATTGATGGGACAGCAACAAGTCTAATTTTCCACGCTACAATGATTCCAAAGTTTTCTCCTCCACCACCACGTATAGCCCAAAACAAATCTTCCCCCATGGATTTTCGATCTAAAACTTTTCCATCAACATTGACTAAGTGCGCATCAATGATATTATCAGCCGCGAGGCCATAATTTCGCATCAATGCTCCATAGCCTCCTCCACTAAAGTGTCCACCCGCGCCAACAGTAGGGCAGTACCCAGCAGGAAAACTAAGATTCTCATTGTTCTCATTGATCCAATAATAAACTTCTCCAAGGGTAGCTCCGGCTTCAACCCATGCAGTTTGGCTATGAACATCTATTTTGACCGAATGCATGTTTCTCAAGTCTACTATAACAAATGGGACTTGAGATATGTAGGACATGCCCTCAGCATCATGACCACCGCTTCGAGTTCGAATCTGCAAGCCAACTTTTTTGGAGCATAGAATAGTGCCTTGGATATGGGAGACATTTAAAGGAGTGATGATAACAAGTGGTTTTGGGGTTGTGTCAGAGGTAAATCTAAGATTTTGTATGGTCGAATTTAGGATAGACATATAAAATTGGTCGTGTTGAGTGTATACGAGTTTTGCATTTGTTACATTGGTGGGAATATATTGTGAGAAGCATTTAAGGAAGTTTTCTTGAGGATTAGCTATTGAAATTTGGATATTGAATGAGAGAAAAAAAAATATTATCTTGCAAACATACC

## Cannbio-2 CBCAS#2 sequence

## ATGAATTGCTCAACATTCTCCTTTTGGTTTGTTTGCAAAATAATATTTTTCTTTCTCTCATTCAATATCCAAATTTCAATAGCTAATCCTCAAGAAAACTTCCTTAAATGCTTCTCGGAATATATTCCTAACAATCCAGCAAATCCAAAATTCATATACACTCAACACGACCAATTGTATATGTCTGTCCTGAATTCGACAATACAAAATCTTAGATTCACCTCTGATACAACCCCAAAACCACTCGTTATTGTCACTCCTTCAAATGTCTCCCATATCCAGGCCAGTATTCTCTGCTCCAAGAAAGTTGGTTTGCAGATTCGAACTCGAAGCGGTGGCCATGATGCTGAGGGTTTGTCCTACATATCTCAAGTCCCATTTGCTATAGTAGACTTGAGAAACATGCATACGGTCAAAGTAGATATTCATAGCCAAACTGCGTGGGTTGAAGCCGGAGCTACCCTTGGAGAAGTTTATTATTGGATCAATGAGATGAATGAGAATTTTAGTTTTCCTGGTGGGTATTGCCCTACTGTTGGCGTAGGTGGACACTTTAGTGGAGGAGGCTATGGAGCATTGATGCGAAATTATGGCCTTGCGGCTGATAATATCATTGATGCACACTTAGTCAATGTTGATGGAAAAGTTCTAGATCGAAAATTCATGGGAGAAGATCTATTTTGGGCTATACGTGGTGGAGGAGGAGAAAACTTTGGAATCATTGCAGCATGGAAAATCAAACTTGTTGTTGTCCCATCAAAGGCTACTATATTCAGTGTTAAAAAGAACATGGAGATACATGGGCTTGTCAAGTTATTTAACAAATGGCAAAATATTGCTTACAAGTATGACAAAGATTTAATGCTCACGACTCACTTCAGAACTAGGAATATTACAGATAATCATGGGAAGAATAAGACTACAGTACATGGTTACTTCTCTTCCATTTTTCTTGGTGGAGTGGATAGTCTAGTTGACTTGATGAACAAGAGCTTTCCTGAGTTGGGTATTAAAAAAACTGATTGCAAAGAATTGAGCTGGATTGATACAACCATCTTCTACAGTGGTGTTGTAAATTACAACACTGCTAATTTTAAAAAGGAAATTTTGCTTGATAGATCAGCTGGGAAGAAGACGGCTTTCTCAATTAAGTTAGACTATGTTAAGAAACTAATACCTGAAACTGCAATGGTCAAAATTTTGGAAAAATTATATGAAGAAGAGGTAGGAGTTGGGATGTATGTGTTGTACCCTTACGGTGGTATAATGGATGAGATTTCAGAATCAGCAATTCCATTCCCTCATCGAGCTGGAATAATGTATGAACTTTGGTACACTGCTACCTGGGAGAAGCAAGAAGATAACGAAAAGCATATAAACTGGGTTCGAAGTGTTTATAATTTCACAACTCCTTATGTGTCCCAAAATCCAAGATTGGCGTATCTCAATTATAGGGACCTTGATTTAGGAAAAACTAATCCTGAGAGTCCTAATAATTACACACAAGCACGTATTTGGGGTGAAAAGTATTTTGGTAAAAATTTTAACAGGTTAGTTAAGGTGAAAACCAAAGCTGATCCCAATAATTTTTTTAGAAACGAACAAAGTATCCCACCTCTTCCACCGCGTCATCAT

## Cannbio-2 CBDAS-truncated#4 sequence (for pRNAi-GG-CBDAS-UNIVERSAL)

TGACGATGCGGTGGAAGAGGTGGGATGCTTTGTTCATTTCTAAAAAAATTATTGGGATCAACCAGGGTTTTCACTTTTACTACCCTGTCAAAATTTTTACCAAAATACTTCTCACCCCAAATACTTGCTTGTGTGTAATTATTTGGACTCTTGGGATCATTTATTCCAGTATCAAGGTCTCTATAATTGAGATATGCCAATCTTGGATTTTAGGACACATAAGGAGTAATGAAGCTATAAACATTTCGAATCCAGTTTAGATGCTTTTTCGTTATCTTCGTGCTTCTCCCAGCTACATATGTACCATAATTCATACATGATTCCAGCTCAATGAGGTAATGGAATTGCTGATTCTGAAATCTCATCCATTATACCACCGTAAGGGTACAACGCATACATCCCAGCTCCTTCATCTTCTTCATATAATTTTTCCAAAATTTTGACAAACGCAGATTCTGGAATTGGTTTCTTAACGTAGTCTAACTTAATCTTTAAAGAACCGTTCTGCCCAGCTGATCTATCAAGCAAAATTTCCTTATTAAAATTATCAGTGCCGTAATTTACAACACCGCTATAAAAGATGATAATATCAATCTAGCTCAATTGTTTGCAATCTGTTTTTTTAATACCCAACTCAGGAAAACTCTTATTCATCAAGTCGACTAGACTATCCACTCCACCAAGGAAAACTGAAGAGAAGTAAGTGTGTATTGTTGTCTTATTCTTCCCATGATTATTTGTAATATTCCTAGTTATGAAGTGAGTCATGAGTAATAAATCTTTGTCATACTTGTAAGCAATATTTTGCCATTTGTTAACTCACTTGACAAGCTCATGTATCTCCATGATCTTTTTAACACTAAACATAGTAGACTTTGTTGGGACAGCAACCAGTCTAATTTTCCATGCTACAATGATTCCGAAGCTTTCTCCTCCACCACCACGTATAGCCCAAAAGAGATCTTCCCCCATAGATTTTCGATCTAGCACTTTTGCATCAACGTTGACTAAGTGTGCATCAACGATATTATCAGCCGCGAGGCCATAATTTTGCATCAATGGTCCATATCCTCCTCCACCAAAGTGTCCAGCTGCGCTAACAGTAGGGCAATACCCAGCAGCCAAACTAAGATTCTCATTTTTCTCATTAACCCAATAATAAACTTCTCCAAGGGTAGCTCCGGCTTCAACCCTTGCGATTTGGCTATGAACATCTATGTTGATTGAATGCATGTTTCTCAAGTCTACTATAACAAATGGGACTTGAGATATGTAGGACATGTCTTCAGAATCATGACCACCGCTTCGAGTTCGAATTTGCAAACCAATTTTCTTGGACATAGAATAGTGCCTTGGATATGAGAGACATGTGAAGGAGTG

## Primers for cDNA amplification

| **Insert** | **Forward Primer** | **Reverse Primer** |
| --- | --- | --- |
| THCAS | accaggtctcaggag AACTATTTTATGCTCTAAGAAAGT | accaggtctcatcgt TCATGACTCACTTCATAACAAA |
| CBDAS-like#1 | accaggtctcaggag AAGTCCCATTTGTTATAGTAGA | accaggtctcatcgt GAGATACATGAGCTTGTCAA |
| CBCAS#2 | accaggtctcaggag GGCCAGTATTCTCTGCTC | accaggtctcatcgt CACGACTCACTTCAGAACTAG |
| CBDAS-UNIVERSAL | accaggtctcaggag CCGGAGCTACCCTT | accaggtctcatcgt GGCTATACGTGGTGG |

## THCAS amplified cDNA insert

AACTATTTTATGCTCTAAGAAAGTTGGCTTGCAGATTCGAACTCGAAGCGGTGGCCATGATGCTGAGGGTATGTCCTACATATCTCAAGTCCCATTTGTTGTAGTAGACTTGAGAAACATGCATTCGATCAAAATAGATGTTCATAGCCAAACTGCGTGGGTTGAAGCCGGAGCTACCCTTGGAGAAGTTTATTATTGGATCAATGAGAAGAATGAGAATCTTAGTTTTCCTGGTGGGTATTGCCCTACTGTTGGCGTAGGTGGACACTTTAGTGGAGGAGGCTATGGAGCATTGATGCGAAATTATGGCCTTGCGGCTGATAATATTATTGATGCACACTTAGTCAATGTTGATGGAAAAGTTCTAGATCGAAAATCCATGGGAGAAGATCTGTTTTGGGCTATACGTGGTGGTGGAGGAGAAAACTTTGGAATCATTGCAGCATGGAAAATCAAACTGGTTGCTGTCCCATCAAAGTCTACTATATTCAGTGTTAAAAAGAACATGGAGATACATGGGCTTGTCAAGTTATTTAACAAATGGCAAAATATTGCTTACAAGTATGACAAAGATTTAGTACTCATGACTCACTTCATAACAAA

## CBDAS-like#1 amplified cDNA insert

AAGTCCCATTTGTTATAGTAGACTTGAGAAACATGCATTCGGTCAAAATAGATGTTCATAGCCAAACTGCATGGGTTGAAGCCGGAGCTACCCTTGGAGAAGTTTATTATTGGATCAATGAGAACAATGAGAATCTTAGTTTTCCTGCTGGGTACTGCCCTACTGTTGGCGCGGGTGGACACTTTAGTGGAGGAGGCTATGGAGCATTGATGCGAAATTATGGCCTCGCGGCTGATAATATCATTGATGCGCACTTAGTCAATGTTGATGGAAAAGTTTTAGATCGAAAATCCATGGGGGAAGATTTGTTTTGGGCTATACGTGGTGGTGGAGGAGAAAACTTTGGAATCATTGTAGCGTGGAAAATTAGACTTGTTGCTGTCCCATCAATGTCTACTATATTCAGTGTTAAAAAGAACATGGAGATACATGAGCTTGTCAA

## CBCAS#2 amplified cDNA insert

GGCCAGTATTCTCTGCTCCAAGAAAGTTGGTTTGCAGATTCGAACTCGAAGCGGTGGCCATGATGCTGAGGGTTTGTCCTACATATCTCAAGTCCCATTTGCTATAGTAGACTTGAGAAACATGCATACGGTCAAAGTAGATATTCATAGCCAAACTGCGTGGGTTGAAGCCGGAGCTACCCTTGGAGAAGTTTATTATTGGATCAATGAGATGAATGAGAATTTTAGTTTTCCTGGTGGGTATTGCCCTACTGTTGGCGTAGGTGGACACTTTAGTGGAGGAGGCTATGGAGCATTGATGCGAAATTATGGCCTTGCGGCTGATAATATCATTGATGCACACTTAGTCAATGTTGATGGAAAAGTTCTAGATCGAAAATTCATGGGAGAAGATCTATTTTGGGCTATACGTGGTGGAGGAGGAGAAAACTTTGGAATCATTGCAGCATGGAAAATCAAACTTGTTGTTGTCCCATCAAAGGCTACTATATTCAGTGTTAAAAAGAACATGGAGATACATGGGCTTGTCAAGTTATTTAACAAATGGCAAAATATTGCTTACAAGTATGACAAAGATTTAATGCTCACGACTCACTTCAGAACTAG

## CBDAS-UNIVERSAL amplified cDNA insert

CCGGAGCTACCCTTGGAGAAGTTTATTATTGGGTTAATGAGAAAAATGAGAATCTTAGTTTGGCTGCTGGGTATTGCCCTACTGTTAGCGCAGCTGGACACTTTGGTGGAGGAGGATATGGACCATTGATGCAAAATTATGGCCTCGCGGCTGATAATATCGTTGATGCACACTTAGTCAACGTTGATGCAAAAGTGCTAGATCGAAAATCTATGGGGGAAGATCTCTTTTGGGCTATACGTGGTGG

# Flanking primers for recombinant E. coli

|  | **Forward Primer** | **Reverse Primer** |
| --- | --- | --- |
| Arm#1 | CATTTGGATTGATTACAGTTGG | ACCCAACTTAATCGCCTT |
| Arm#2 | CGAATCTCAAGCAATCAAGC | ATTTTGAGGCATTTCAGTCA |

# Primer sequences for qPCR

| **Insert** | **Forward Primer** | **Reverse Primer** |
| --- | --- | --- |
| THCAS | AATTTTTCATGGTGGAGTGGAT | AAAATTTACAACACCACTGTAGAA |
| CBDAS | CAGTTACTTCTCCTCCATTTTCC | ATAGTATCAATCCAGCTCAACT |
| CBCAS | ACCTGGGAGAAGCAAGAAG | GGATTAGTTTTTCCTAAATCAAGG |

## pRNAi-GG vectors designed for study

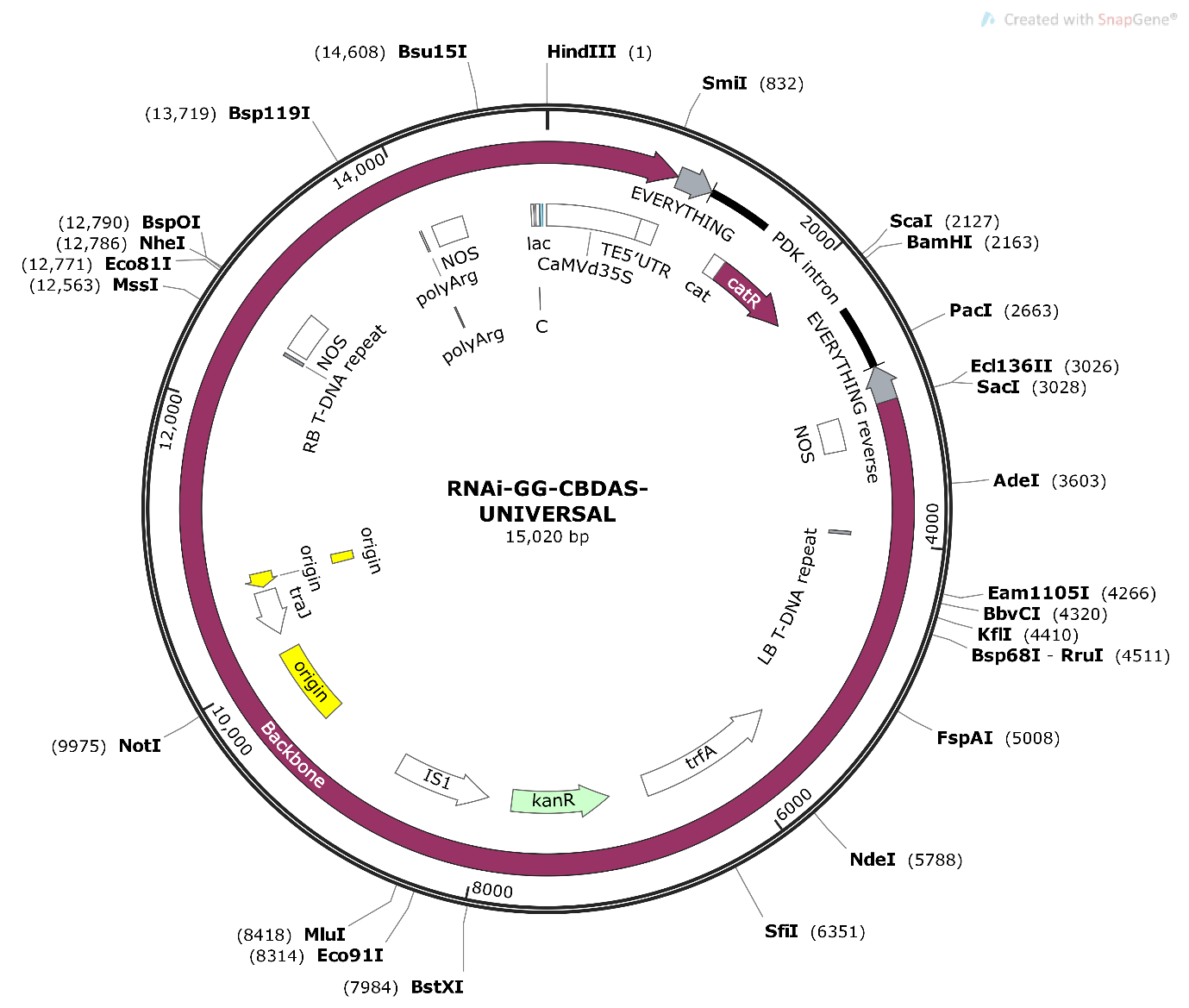


## Global alignment of all cannabinoid biosynthesis genes coding sequences

Consensus ATGTCTRTCCTGAATTCGACAATACAAAATCTTAGATTCACCTCTGATACAACCCCAAAA 60

THCAS ATGTCTATCCTGAATTCGACAATACAAAATCTTAGATTCATCTCTGATACAACCCCAAAA 60

CBCAS#1 ATGTCTGTCCTGAATTCGACAATACAAAATCTTAGATTCACCTCTGATACAACCCCAAAA 60

CBCAS#2 ATGTCTGTCCTGAATTCGACAATACAAAATCTTAGATTCACCTCTGATACAACCCCAAAA 60

CBCAS-truncated ATGTCTGTCCTGAATTCGACAATACAAAATCTTAGATTCACCTCTGATACAACCCCAAAA 60

CBDAS#1 ------------------------------------------------------------

CBDAS#2 ------------------------------------------------------------

CBDAS-like#1 ------------------------------------------------------------

CBDAS-like#2 ATGTCTATCTTAAATTCGACCATACAAAATCTTAGATTTACCTCTGACACAACCCCAAAA 60

CBDAS-like#3 ATGTCTATCTTAAATTCGACCGTACAAAATCTTAGATTTACCTCTGACACAACCCCAAAA 60

CBDAS-truncated#1 ------------------------------------------------------------

CBDAS-truncated#2 ------------------------------------TTTACCTCTGACACAACCCCAAAA 24

CBDAS-truncated#3 ------------------------------------------------------------

CBDAS-truncated#4 ------------------------------------------------------------

Consensus CCACTCGTTATTGTCACTCCTTCAAATGTCTCCCATATCCAAGCCACTATTCTATGCTCC 120

THCAS CCACTCGTTATTGTCACTCCTTCAAATAACTCCCATATCCAAGCAACTATTTTATGCTCT 120

CBCAS#1 CCACTCGTTATTGTCACTCCTTCAAATGTCTCCCATATCCAGGCCAGTATTCTCTGCTCC 120

CBCAS#2 CCACTCGTTATTGTCACTCCTTCAAATGTCTCCCATATCCAGGCCAGTATTCTCTGCTCC 120

CBCAS-truncated CCACTCGTTATTGTCACTCCTTCAAATGTCTCCCATATCCAGGCCAGTATTCTCTGCTCC 120

CBDAS#1 ------------------------------------------------------------

CBDAS#2 ------------------------------------------------------------

CBDAS-like#1 ------------------------------------------------------------

CBDAS-like#2 CCACTTGTTATCATCACTCCTTTAAATGTCTCCCATATCCAAGGCACTATTCTATGCTCC 120

CBDAS-like#3 CCACTTGTTATCACCACTCCTTTAAATGTCTCCCATATCCAAGGCACTATTCTATGTTCC 120

CBDAS-truncated#1 ------------------------------------------------------------

CBDAS-truncated#2 CCACTTGTTATCATCGCTCCTTTAAATGTCTCCCATATCCAAGGCACTATTCTATGCTCC 84

CBDAS-truncated#3 ------------------------------------------------------------

CBDAS-truncated#4 -----------------------------------------------------ATG-TCC 6

Consensus AAGAAAGTTGGYTTGCAGATTCGAACTCGAAGCGGTGGTCATGATGCTGAGGGCATGTCC 180

THCAS AAGAAAGTTGGCTTGCAGATTCGAACTCGAAGCGGTGGCCATGATGCTGAGGGTATGTCC 180

CBCAS#1 AAGAAAGTTGGTTTGCAGATTCGAACTCGAAGCGGTGGCCATGATGCTGAGGGTTTGTCC 180

CBCAS#2 AAGAAAGTTGGTTTGCAGATTCGAACTCGAAGCGGTGGCCATGATGCTGAGGGTTTGTCC 180

CBCAS-truncated AAGAAAGTTGGTTTGCAGATTCGAACTCGAAGCGGTGGCCATGATGCTGAGGGTTTGTCC 180

CBDAS#1 ------------------------------------------------------ATGTCC 6

CBDAS#2 ------------------------------------------------------ATGTCC 6

CBDAS-like#1 ------------------ATTCGAACTCGAAGCGGTGGTCATGATGCTGAGGGCATGTCC 42

CBDAS-like#2 AAGAAAGTTGGCTTGCAGATTCGAACTCGAAGCGGTGGTCATGATGCTGAGGGCATGTCC 180

CBDAS-like#3 AAGAAAGTTGGCTTGCAGATTCGAACTCGAAGCGGTGGTCATGATGCTGAGGGCATGTCC 180

CBDAS-truncated#1 ------------------------------------------------------------

CBDAS-truncated#2 AAGAAAGTTGGCTTGCAGATTCGAGCTCGAAGCAGTGGTCATGATGCTGAGGGCATGTCC 144

CBDAS-truncated#3 ------------------------------------------------------------

CBDAS-truncated#4 AAGAAAATTGGTTTGCAAATTCGAACTCGAAGCGGTGGTCATGATTCTGAAGACATGTCC 66

Consensus TACATATCTCAAGTCCCATTTGTTATAGTAGACTTGAGAAACATGCATTCGGTCAAAATA 240

THCAS TACATATCTCAAGTCCCATTTGTTGTAGTAGACTTGAGAAACATGCATTCGATCAAAATA 240

CBCAS#1 TACATATCTCAAGTCCCATTTGCTATAGTAGACTTGAGAAACATGCATACGGTCAAAGTA 240

CBCAS#2 TACATATCTCAAGTCCCATTTGCTATAGTAGACTTGAGAAACATGCATACGGTCAAAGTA 240

CBCAS-truncated TACATATCTCAAGTCCCATTTGCTATAGTAGACTTGAGAAACATCCATACGGTCAAAGTA 240

CBDAS#1 TACATATCTGAAGTCCCATTTGTTATAGTAGACTTGAGAAACATGCATTCGGTCAAAATA 66

CBDAS#2 TACATATCTGAAGTCCCATTTGTTATAGTAGACTTGAGAAACATGCATTCGGTCAAAATA 66

CBDAS-like#1 TACATATCTCAAGTCCCATTTGTTATAGTAGACTTGAGAAACATGCATTCGGTCAAAATA 102

CBDAS-like#2 TACATATCTCAAGTCCCATTTGTTATAGTAGACTTGAGAAACATGCATTCGGTCAAAATA 240

CBDAS-like#3 TACATATCTCAAGTCCCATTTGTTATAGTAGACTTGAGAAACATGCATTCGGTCAAAATA 240

CBDAS-truncated#1 ------------------------------------------------------------

CBDAS-truncated#2 TACATATCTCAAGTCCCATTTGTTATAGTAGACTTGAGAAACATGCATTCGGTCAAAATA 204

CBDAS-truncated#3 ------------------------------------------------------------

CBDAS-truncated#4 TACATATCTCAAGTCCCATTTGTTATAGTAGACTTGAGAAACATGCATTCAATCAACATA 126

Consensus GATGTTCATAGCCAAACTGCATGGGTTGAAGCCGGAGCTACCCTTGGAGAAGTTTATTAT 300

THCAS GATGTTCATAGCCAAACTGCGTGGGTTGAAGCCGGAGCTACCCTTGGAGAAGTTTATTAT 300

CBCAS#1 GATATTCATAGCCAAACTGCGTGGGTTGAAGCCGGAGCTACCCTTGGAGAAGTTTATTAT 300

CBCAS#2 GATATTCATAGCCAAACTGCGTGGGTTGAAGCCGGAGCTACCCTTGGAGAAGTTTATTAT 300

CBCAS-truncated GATATTCATAGCCAAACTGCGTGGGTTGAAGCCGGAGCTACCCTTGGAGAAGTTTATTAT 300

CBDAS#1 GATGTTCATAGCCAAACTGCATGGGTTGAAGCCGGAGCTACCCTTGGAGAAGTTTATTAT 126

CBDAS#2 GATGTTCATAGCCAAACTGCATGGGTTGAAGCCGGAGCTACCCTTGGAGAAGTTTATTAT 126

CBDAS-like#1 GATGTTCATAGCCAAACTGCATGGGTTGAAGCCGGAGCTACCCTTGGAGAAGTTTATTAT 162

CBDAS-like#2 GATGTTCATAGCCAAAATGCATGGGTTGAAGCCGGAGCTACCCTTGGAGAAGTTTATTAT 300

CBDAS-like#3 GATGTTCATAGCCAAACTGCATGGGTTGAATCCGGAGCTACCCTTGGAGAAGTTTATTAT 300

CBDAS-truncated#1 ------------------------------------------------------------

CBDAS-truncated#2 GATGTTCATAGCCAAACTGCATGGGTTGAAGCCGGAGCTACCCTTGGAGAAGTTTATTAT 264

CBDAS-truncated#3 ------------------------------------------------------------

CBDAS-truncated#4 GATGTTCATAGCCAAATCGCAAGGGTTGAAGCCGGAGCTACCCTTGGAGAAGTTTATTAT 186

Consensus TGGATCAATGAGAACAATGAGAATCTTAGTTTTCCTGCTGGGTACTGCCCTACTGTTGGC 360

THCAS TGGATCAATGAGAAGAATGAGAATCTTAGTTTTCCTGGTGGGTATTGCCCTACTGTTGGC 360

CBCAS#1 TGGATCAATGAGATGAATGAGAATTTTAGTTTTCCTGGTGGGTATTGCCCTACTGTTGGC 360

CBCAS#2 TGGATCAATGAGATGAATGAGAATTTTAGTTTTCCTGGTGGGTATTGCCCTACTGTTGGC 360

CBCAS-truncated TGGATCAATGAGATGAATGAGAATTTTAGTTTTCCTGGTGGGTATTGCCCTACTGTTGGC 360

CBDAS#1 TGGATCAATGAGAACAATGAGAATCTTAGTTTTCCTGCTGGGTACTGCCCTACTGTTGGC 186

CBDAS#2 TGGATCAATGAGAACAATGAGAATCTTAGTTTTCCTGCTGGGTACTGCCCTACTGTTGGC 186

CBDAS-like#1 TGGATCAATGAGAACAATGAGAATCTTAGTTTTCCTGCTGGGTACTGCCCTACTGTTGGC 222

CBDAS-like#2 TGGATCAATGAGAACAATGAGAATCTTAGTTTTCCTGCTGGGTACTGCCCTACTGTTGGC 360

CBDAS-like#3 TGGATCAATGAGAACAATGAGAATCTTAGTTTTCCTGCTGGGTACTGCCCTACTGTTGGC 360

CBDAS-truncated#1 ------------------------------------------------------------

CBDAS-truncated#2 TGGATCAATGAGAACAATGAGAATCTTAGTTTTCCTGCTGGGTACTGTCCTACTGTTGGC 324

CBDAS-truncated#3 ------------------------------------------------------------

CBDAS-truncated#4 TGGGTTAATGAGAAAAATGAGAATCTTAGTTTGGCTGCTGGGTATTGCCCTACTGTTAGC 246

Consensus GCRGGTGGACACTTTAGTGGAGGAGGCTATGGAGCATTGATGCGAAATTATGGCCTCGCG 420

THCAS GTAGGTGGACACTTTAGTGGAGGAGGCTATGGAGCATTGATGCGAAATTATGGCCTTGCG 420

CBCAS#1 GTAGGTGGACACTTTAGTGGAGGAGGCTATGGAGCATTGATGCGAAATTATGGCCTTGCG 420

CBCAS#2 GTAGGTGGACACTTTAGTGGAGGAGGCTATGGAGCATTGATGCGAAATTATGGCCTTGCG 420

CBCAS-truncated GTAGGTGGACACTTTAGTGGAGGAGGCTATGGAGCATTGATGCGAAATTATGGCCTTGCG 420

CBDAS#1 GCGGGTGGACACTTTAGTGGAGGAGGCTATGGAGCATTGATGCGAAATTATGGCCTCGCG 246

CBDAS#2 GCGGGTGGACACTTTAGTGGAGGAGGCTATGGAGCATTGATGCGAAATTATGGCCTCGCG 246

CBDAS-like#1 GCGGGTGGACACTTTAGTGGAGGAGGCTATGGAGCATTGATGCGAAATTATGGCCTCGCG 282

CBDAS-like#2 GCTGGTGGACACTTTAGTGGAGGAGGCTATGGAGCATTGATGCGAAATTATGGCCTCGCG 420

CBDAS-like#3 ACGGGTGGACACTTTAGTGGAGGAGGCTATGGAGCATTGATGCGAAATTATGGCCTCGCG 420

CBDAS-truncated#1 ------------------------------------------------------------

CBDAS-truncated#2 GCGGGTGGACACTTTAGTGGAGGAGGCTATGGAGCATTGATGCGAAATTATGGCCTCGCG 384

CBDAS-truncated#3 ------------------------------------------------------------

CBDAS-truncated#4 GCAGCTGGACACTTTGGTGGAGGAGGATATGGACCATTGATGCAAAATTATGGCCTCGCG 306

Consensus GCTGATAATATCATTGATGCACACTTAGTCAATGTTGATGGAAAAGTTTTAGATCGAAAA 480

THCAS GCTGATAATATTATTGATGCACACTTAGTCAATGTTGATGGAAAAGTTCTAGATCGAAAA 480

CBCAS#1 GCTGATAATATCATTGATGCACACTTAGTCAATGTTGATGGAAAAGTTCTAGATCGAAAA 480

CBCAS#2 GCTGATAATATCATTGATGCACACTTAGTCAATGTTGATGGAAAAGTTCTAGATCGAAAA 480

CBCAS-truncated GCTGATAATATCATTGATGCACACTTAGTCAATGTTGATGGAAAAGTTCTAGATCGAAAA 480

CBDAS#1 GCTGATAATATCATTGATGCGCACTTAGTCAATGTTGATGGAAAAGTTTTAGATCGAAAA 306

CBDAS#2 GCTGATAATATCATTGATGCGCACTTAGTCAATGTTGATGGAAAAGTTTTAGATCGAAAA 306

CBDAS-like#1 GCTGATAATATCATTGATGCGCACTTAGTCAATGTTGATGGAAAAGTTTTAGATCGAAAA 342

CBDAS-like#2 GCTGATAATATCATTGATGCACACTTAGTCAATGTTGATGGAAAAGTTTTAGATCGAAAA 480

CBDAS-like#3 GCTGATAATATCATTGATGCGCACTTAGTCAATGTTGATGGAAAAGTTTTAGATCGAAAA 480

CBDAS-truncated#1 ------------------------------------------------------------

CBDAS-truncated#2 GCTGATAATATCATTGATGCGCACTTAGTCAATGTTGATGGAAAAGTTTTAGATCGAAAA 444

CBDAS-truncated#3 ------------------------------------------------------------

CBDAS-truncated#4 GCTGATAATATCGTTGATGCACACTTAGTCAACGTTGATGCAAAAGTGCTAGATCGAAAA 366

Consensus TCCATGGGGGAAGATYTGTTTTGGGCTATACGTGGTGGTGGAGGAGAAAACTTTGGAATC 540

THCAS TCCATGGGAGAAGATCTGTTTTGGGCTATACGTGGTGGTGGAGGAGAAAACTTTGGAATC 540

CBCAS#1 TCCATGGGAGAAGATCTATTTTGGGCTATACGTGGTGGAGGAGGAGAAAACTTTGGAATC 540

CBCAS#2 TTCATGGGAGAAGATCTATTTTGGGCTATACGTGGTGGAGGAGGAGAAAACTTTGGAATC 540

CBCAS-truncated TCCATGGGAGAAGATCTATTTTGGGCTATACGTGGTGGAGGAGGAGAAAACTTTGGAATC 540

CBDAS#1 TCCATGGGGGAAGATTTGTTTTGGGCTATACGTGGTGGTGGAGGAGAAAACTTTGGAATC 366

CBDAS#2 TCCATGGGGGAAGATTTGTTTTGGGCTATACGTGGTGGTGGAGGAGAAAACTTTGGAATC 366

CBDAS-like#1 TCCATGGGGGAAGATTTGTTTTGGGCTATACGTGGTGGTGGAGGAGAAAACTTTGGAATC 402

CBDAS-like#2 TCCATGGGGGAAGATTTGTTTTGGGCTATACGTGGTGGTGGAGGAGAAAACTTTGGAATC 540

CBDAS-like#3 TCCATGGGGGAAGATTTGTTTTGGGCTATACGTGGTGGTGGAGGAGAAAACTTTGGAATC 540

CBDAS-truncated#1 ------------------------------------------------------------

CBDAS-truncated#2 TCCATGGGGGAAGATTTGTTTTGGGCTATACGTGGTGGTGGAGGAGAAAACTCTGGAATC 504

CBDAS-truncated#3 ---ATGGGACAAGATCTCTTTTGGGCTATACGTGGTGGTGGAAGAGAAAGCTTCAGAATC 57

CBDAS-truncated#4 TCTATGGGGGAAGATCTCTTTTGGGCTATACGTGGTGGTGGAGGAGAAAGCTTCGGAATC 426

Consensus ATTGCAGCRTGGAAAATTAGACTTGTTGCTGTCCCATCAAWGTCTACTATATTCAGTGTT 600

THCAS ATTGCAGCATGGAAAATCAAACTGGTTGCTGTCCCATCAAAGTCTACTATATTCAGTGTT 600

CBCAS#1 ATTGCAGCATGGAAAATCAAACTTGTTGTTGTCCCATCAAAGGCTACTATATTCAGTGTT 600

CBCAS#2 ATTGCAGCATGGAAAATCAAACTTGTTGTTGTCCCATCAAAGGCTACTATATTCAGTGTT 600

CBCAS-truncated ATTGCAGCATGGAAAATCAAACTTGTTGTTGTCCCATCAAAGGCTACTATATTCAGTGTT 600

CBDAS#1 ATTGCAGCGTGGAAAATTAGACTTGTTGCTGTCCCATCAATGTCTACTATATTCAGTGTT 426

CBDAS#2 ATTGCAGCGTGGAAAATTAGACTTGTTGCTGTCCCATCAATGTCTACTATATTCAGTGTT 426

CBDAS-like#1 ATTGTAGCGTGGAAAATTAGACTTGTTGCTGTCCCATCAATGTCTACTATATTCAGTGTT 462

CBDAS-like#2 ATTGCAGCGTGGAAAATTAGACTTGTTGCTGTCCCATCAATGTCTACTATATTCAGTGTT 600

CBDAS-like#3 ATTGCAGCGTGGAAAATTAGACTTGTTGCTGTCCCATCAATGTCTACTATATTCAGTGTT 600

CBDAS-truncated#1 ------------------------------------------------------------

CBDAS-truncated#2 ATTGCAGCGTGGAAAATTAGACTTGTTGCTGTCCCATCAATGTCTACTATATTCAGTGTT 564

CBDAS-truncated#3 ATTGTAGCATGGAAAATTAGACTGGTTGCTGTCCCAACAAAGTCTACTATGTTTAGTGTT 117

CBDAS-truncated#4 ATTGTAGCATGGAAAATTAGACTGGTTGCTGTCCCAACAAAGTCTACTATGTTTAGTGTT 486

Consensus AAAAAGAACATGGAGATACATGAGCTTGTCAAGTTAGTTAACAAATGGCAAAATATTGCT 660

THCAS AAAAAGAACATGGAGATACATGGGCTTGTCAAGTTATTTAACAAATGGCAAAATATTGCT 660

CBCAS#1 AAAAAGAACATGGAGATACATGGGCTTGTCAAGTTATTTAACAAATGGCAAAATATTGCT 660

CBCAS#2 AAAAAGAACATGGAGATACATGGGCTTGTCAAGTTATTTAACAAATGGCAAAATATTGCT 660

CBCAS-truncated AAAAAGAACATGGAGATACATGGGCTTGTCAAGTTATTTAACAAATGGCAAAATATTGCT 660

CBDAS#1 AAAAAGAACATGGAGATACATGAGCTTGTCAAGTTAGTTAACAAATGGCAAAATATTGCT 486

CBDAS#2 AAAAAGAACATGGAGATACATGAGCTTGTCAAGTTAGTTAACAAATGGCAAAATATTGCT 486

CBDAS-like#1 AAAAAGAACATGGAGATACATGAGCTTGTCAAGTTAGTTAACAAATGGCAAAATATTGCT 522

CBDAS-like#2 AAAAAGAACATGGAGATACATGAGCTTGTCAAGTTAGTTAACAAATGGCAAAATATTGCT 660

CBDAS-like#3 AAAAAGAACATGGAGATACATGAGCTTGTCAAGTTAGTTAACAAATGGCAAAATATTGCT 660

CBDAS-truncated#1 ------------------------------------------------------------

CBDAS-truncated#2 AAAAAGAACATGGAGATACATGAGCTTGTCAA---------------------------- 596

CBDAS-truncated#3 AAAAAGATCAAGGAGATACATGAGCTTGTGAAGTTAGTTAACAAGTGGCAAAATATTTCT 177

CBDAS-truncated#4 AAAAAGATCATGGAGATACATGAGCTTGTCAA---------------------------- 518

Consensus TACAWGTATGAMAAAGAWTTATTACTCWTKACTCACTTYATAACCAGGAATATTACAGAT 720

THCAS TACAAGTATGACAAAGATTTAGTACTCATGACTCACTTCATAACAAAGAATATTACAGAT 720

CBCAS#1 TACAAGTATGACAAAGATTTAATGCTCACGACTCACTTCAGAACTAGGAATATTACAGAT 720

CBCAS#2 TACAAGTATGACAAAGATTTAATGCTCACGACTCACTTCAGAACTAGGAATATTACAGAT 720

CBCAS-truncated TACAAGTATGACAAAGATTTAATGCTCACGACTCACTTCAGAACTAGGAATATTACAGAT 720

CBDAS#1 TACATGTATGAAAAAGAATTATTACTCTTTACTCACTTTATAACCAGGAATATTACAGAT 546

CBDAS#2 TACATGTATGAAAAAGAATTATTACTCTTTACTCACTTTATAACCAGGAATATTACAGAT 546

CBDAS-like#1 TACATGTATGAAAAAGAATTATTACTCTTTACTCACTTTATAACCAGGAATATTACAGAT 582

CBDAS-like#2 TACATGTATGAAAAAGAATTATTACTCTTTACTCACTTTATAACCAGGAATATTACAGAT 720

CBDAS-like#3 TACATGTATGAAAAAGAATTATTACTCTTTACTCACTTTATAACCAGGAATATTACAGAT 720

CBDAS-truncated#1 ------------------------------------------------------------

CBDAS-truncated#2 ------------------------------------------------------------ 596

CBDAS-truncated#3 TACAAGTATGACATAGATTTATTACTCATGACTCACTTCATAACTAGGAATATTACAGAT 237

CBDAS-truncated#4 ------------------------------------------------------------ 518

Consensus AATCAAGGGAAGAATAAGACAACAATACACAGTTACTTCTCYTCCATTTTCC-NTGGTGG 779

THCAS AATCATGGGAAGAATAAGACTACAGTACATGGTTACTTCTCTTCAATTTTTC-ATGGTGG 779

CBCAS#1 AATCATGGGAAGAATAAGACTACAGTACATGGTTACTTCTCTTCCATTTTTCT-TGGTGG 779

CBCAS#2 AATCATGGGAAGAATAAGACTACAGTACATGGTTACTTCTCTTCCATTTTTCT-TGGTGG 779

CBCAS-truncated AATCATGGGAAGAATAAGACTACAGTACATGGTTACTTCTCTTCCATTTTTCT-TGGTGG 779

CBDAS#1 AATCAAGGGAAGAATAAGACAACAATACACAGTTACTTCTCCTCCATTTTCC-ATGGTGG 605

CBDAS#2 AATCAAGGGAAGAATAAGACAACAATACACAGTTACTTCTCCTCCATTTTCC-ATGGTGG 605

CBDAS-like#1 AATCAAGGGAAGAATAAGACAACAATACACAGTTACTTCTCCTCCATTTTCC-ATGGTGG 641

CBDAS-like#2 AATCAAGGGAAGAATAAGACAACAATACACAGTTACTTCTCCTCCATTTTCC-ATGGTGG 779

CBDAS-like#3 AATCAAGGGAAGAATAAGACAACAATACACAGTTACTTCTCCTCCATTTTCC-ATGGTGG 779

CBDAS-truncated#1 ------------------------------------------------------------

CBDAS-truncated#2 ----------A------------------------------------------------- 597

CBDAS-truncated#3 AATCAAGGGAAGAATAAGACAACAATACACACTTACTTCTCTTTAGTTTTCCT-TGGTGG 296

CBDAS-truncated#4 -------------------C---------------------------------------- 519

Consensus AGTGGATAGTCTAGTCGACTTGATGAACAAGAGCTTTCCTGARTTGGGTATTAAAAAAAC 839

THCAS AGTGGATAGTCTAGTCGACTTGATGAACAAGAGCTTTCCTGAGTTGGGTATTAAAAAAAC 839

CBCAS#1 AGTGGATAGTCTAGTTGACTTGATGAACAAGAGCTTTCCTGAGTTGGGTATTAAAAAAAC 839

CBCAS#2 AGTGGATAGTCTAGTTGACTTGATGAACAAGAGCTTTCCTGAGTTGGGTATTAAAAAAAC 839

CBCAS-truncated AGTGGATAGTCTAGTTGACTTGATGAACAAGAGCTTTCCTGAGTTGGGTATTAAAAAAAC 839

CBDAS#1 AGTGGATAGTCTAGTCGACTTGATGAACAAGAGCTTTCCTGAATTGGGTATTAAAAAAAC 665

CBDAS#2 AGTGGATAGTCTAGTCGACTTGATGAACAAGAGCTTTCCTGAATTGGGTATTAAAAAAAC 665

CBDAS-like#1 AGTGGATAGTCTAGTCGACTTGATGAACAAGAGCTTTCCTGAATTGGGTATTAAAAAAAT 701

CBDAS-like#2 AGTGGATAGTCTAGTCGACTTGATGAACAAGAGCTTTCCTGAATTGGGTATTAAAAAAAG 839

CBDAS-like#3 AGTGGATAGTCTAGTCGACTTGATGAACAAGAGCTTTCCTGAATTGGGTATTAAAAAAAA 839

CBDAS-truncated#1 ------------------------------------------------------------

CBDAS-truncated#2 ------------------------------------------------------------ 597

CBDAS-truncated#3 AGTCGATAGTCTAGTCGACTTAATGAACAAGAGTTTTCCTGAGTTTGGTATTAAAAAAAT 356

CBDAS-truncated#4 ----------------------------------------G------------------- 520

Consensus AGATTGCAAACARTTGAGCTGGATTGATACTATCATCTTCTACAGTGGTGTTGTAAATTA 899

THCAS TGATTGCAAAGAATTTAGCTGGATTGATACAACCATCTTCTACAGTGGTGTTGTAAATTT 899

CBCAS#1 TGATTGCAAAGAATTGAGCTGGATTGATACAACCATCTTCTACAGTGGTGTTGTAAATTA 899

CBCAS#2 TGATTGCAAAGAATTGAGCTGGATTGATACAACCATCTTCTACAGTGGTGTTGTAAATTA 899

CBCAS-truncated TGATTGCAAAGAATTGAGCTGGATTGATACAACCATCTTCTACAGTGGTGTTGTAAATTA 899

CBDAS#1 AGATTGCAAACAGTTGAGCTGGATTGATACTATCATCTTCTACAGTGGTGTTGTAAATTA 725

CBDAS#2 AGATTGCAAACAGTTGAGCTGGATTGATACTATCATCTTCTACAGTGGTGTTGTAAATTA 725

CBDAS-like#1 AGATTGCAAACAGTTGAGCTGGATTGATACTATCATCTTCTACAGTGGTCTTGTAAATTA 761

CBDAS-like#2 AGATTGCAAACAGTTGAGCTGGATTGATACTATCATCTTCTACAGTGGTCTTGTAAATTA 899

CBDAS-like#3 AGATTGCAAACAGTTGAGCTGGATTGATACTATCATCTTCTACAGTGGTGTTGTAAATTA 899

CBDAS-truncated#1 ------------------------------------------------------------

CBDAS-truncated#2 ------------------------------------------------------------ 597

CBDAS-truncated#3 AGATTGCAAACAATTGAGCTGGATTGATACTATCATCTTCTATAGCGGTGTTGTAAATTA 416

CBDAS-truncated#4 -------------------------G----------------------TGTTGTAAATTA 533

Consensus CAACACTGCTAATTTTAAAAARGAAATTTTGCTTGATAGATCAGSTGGGCRGAAGGCGGC 959

THCAS TAACACTGCTAATTTTAAAAAGGAAATTTTGCTTGATAGATCAGCTGGGAAGAAGACGGC 959

CBCAS#1 CAACACTGCTAATTTTAAAAAGGAAATTTTGCTTGATAGATCAGCTGGGAAGAAGACGGC 959

CBCAS#2 CAACACTGCTAATTTTAAAAAGGAAATTTTGCTTGATAGATCAGCTGGGAAGAAGACGGC 959

CBCAS-truncated CAACACTGCTAATTTTAAAAAGGAAATTTTGCTTGATAGATCAGCTGGGAAGAAGACGGC 959

CBDAS#1 CAACACAACTAATTTTAAAAAAGAAATTTTGCTTGATAGATCAGGTGGGCGGAAGGCGGC 785

CBDAS#2 CAACACAACTAATTTTAAAAAAGAAATTTTGCTTGATAGATCAGGTGGGCGGAAGGCGGC 785

CBDAS-like#1 CAACACAACTAATTTTAAAAAAGAAATTTTGCTTGATAGATCAGGTGGGCGGAAGGCGGC 821

CBDAS-like#2 CAACACAACTAATTTTAAAAAAGAAATTTTGCTTGATAGATCAGGTGGGCGGAAGGCGGC 959

CBDAS-like#3 CAACACAACTAATTTTAAAAAAGAAATTTTGCTTGATAGATCAGGTGGGCGGAAGGCGGC 959

CBDAS-truncated#1 ------------------------------------------------------------

CBDAS-truncated#2 ----------------------------------------TCAGGTGGGCGGAAGGCGGC 617

CBDAS-truncated#3 CGGCACTGATAATTTTAATAACCAAATTTCGCTTGTTAGATCAGCTGGGCAGAACGGTGC 476

CBDAS-truncated#4 CGGCACTGATAATTTTAATAAGGAAATTTTGCTTGATAGATCAGCTGGGCAGAACGGTTC 593

Consensus TTTCTCGATTAAGTTAGACTATGTTAAGAAACCGATTCCAGAAACCGCAATGGTCACAAT 1019

THCAS TTTCTCAATTAAGTTAGACTATGTTAAGAAACCAATTCCAGAAACTGCAATGGTCAAAAT 1019

CBCAS#1 TTTCTCAATTAAGTTAGACTATGTTAAGAAACTAATACCTGAAACTGCAATGGTCAAAAT 1019

CBCAS#2 TTTCTCAATTAAGTTAGACTATGTTAAGAAACTAATACCTGAAACTGCAATGGTCAAAAT 1019

CBCAS-truncated TTTCTCAATT-------------------------------------------------- 1019

CBDAS#1 TTTCTCGATTAAGTTAGACTATGTTAAGAAACCGATTCCAGAAACCGCAATGGTCACAAT 845

CBDAS#2 TTTCTCGATTAAGTTAGACTATGTTAAGAAACCGATTCCAGAAACCGCAATGGTCACAAT 845

CBDAS-like#1 TTTCTCGATTAAGTTAGACTATGTTAAGAAACCGATTCCAGAAACCGCAATGGTCACAAT 881

CBDAS-like#2 TTTCTCGATTAAGTTAGACTATGTTAAGAAACCGATTCCAGAAACCGCAATGGTCACAAT 1019

CBDAS-like#3 TTTCTCGATTAAGTTAGACTATGTTAAGAAACCGATTCCAGAAACCGCAATGGTCACAAT 1019

CBDAS-truncated#1 -------------------------------------------------ATGGTCACAAT 11

CBDAS-truncated#2 TTTCTCAATTAAGTTAGACTATGTTAAGAAACCGATTCCAGAAACCGCAATGGTCACAAT 677

CBDAS-truncated#3 TTTCAAGATTAAGTTAGACTATGTTAAGAAACCAATTCCAGAATCTGCATTTGTCAAAAT 536

CBDAS-truncated#4 TTTAAAGATTAAGTTAGACTACGTTAAGAAACCAATTCCAGAATCTGCGTTTGTCAAAAT 653

Consensus TTTGGAAAAATTATATGAAGAAGATGTAGGAGTTGGGATGTTTGTGTTTTACCCTTATGG 1079

THCAS TTTGGAAAAATTATATGAAGAAGATGTAGGAGCTGGGATGTATGTGTTGTACCCTTACGG 1079

CBCAS#1 TTTGGAAAAATTATATGAAGAAGAGGTAGGAGTTGGGATGTATGTGTTGTACCCTTACGG 1079

CBCAS#2 TTTGGAAAAATTATATGAAGAAGAGGTAGGAGTTGGGATGTATGTGTTGTACCCTTACGG 1079

CBCAS-truncated ------------------------------------------------------------ 1079

CBDAS#1 TTTGGAAAAATTATATGAAGAAGATGTAGGAGTTGGGATGTTTGTGTTTTACCCTTATGG 905

CBDAS#2 TTTGGAAAAATTATATGAAGAAGATGTAGGAGTTGGGATGTTTGTGTTTTACCCTTATGG 905

CBDAS-like#1 TTTGGAAAAATTATATGAAGAAGATGTAGGAGTTGGGATGTTTGTGTTTTACCCTTATGG 941

CBDAS-like#2 TTTGGAAAAATTATATGAAGAAGATGTAGGAGTTGGGATGTTTGTGTTTTACCCTTATGG 1079

CBDAS-like#3 TTTGGAAAAATTATATGAAGAAGATGTAGGAGTTGGGATGTTTGTGTTTTACCCTTATGG 1079

CBDAS-truncated#1 TTTGGAAAAATTATATGAAGAAGATGTAGGAGTTGGGATGTTTGTGTTTTACCCTTATGG 71

CBDAS-truncated#2 TTTGGAAAAATTATATGAAGAAGATGTAGGAGTTGGGATGTTCGTGTTTTACCCTTATGG 737

CBDAS-truncated#3 TTTGGAAAAATTATATGAAGAAGATAAAGGAGTTGGGATGTATGCGTTGTACCCTTACGG 596

CBDAS-truncated#4 TTTGGAAAAATTATATGAAGAAGATGAAGGAGCTGGGATGTATGCGTTGTACCCTTACGG 713

Consensus TGGTATAATGGATGAGATTTCAGAATCAGCAATTCCATTCCCTCATCGAGCTGGAATCAT 1139

THCAS TGGTATAATGGAGGAGATTTCAGAATCAGCAATTCCATTCCCTCATCGAGCTGGAATAAT 1139

CBCAS#1 TGGTATAATGGATGAGATTTCAGAATCAGCAATTCCATTCCCTCATCGAGCTGGAATAAT 1139

CBCAS#2 TGGTATAATGGATGAGATTTCAGAATCAGCAATTCCATTCCCTCATCGAGCTGGAATAAT 1139

CBCAS-truncated ------------------------------------------------------------ 1139

CBDAS#1 TGGTATAATGGATGAGATTTCAGAATCAGCAATTCCATTCCCTCATCGAGCTGGAATCAT 965

CBDAS#2 TGGTATAATGGATGAGATTTCAGAATCAGCAATTCCATTCCCTCATCGAGCTGGAATCAT 965

CBDAS-like#1 TGGTATAATGGATGAGATTTCAGAATCAGCAATTCCATTCCCTCATCGAGCTGGAATCAT 1001

CBDAS-like#2 TGGTATAATGGATGAGATTTCAGAATCAGCAATTCCATTCCCTCATCGAGCTGGAATCAT 1139

CBDAS-like#3 TGGTATAATGGATGAGATTTCAGAATCAGCAATTCCATTCCCTCATCGAGCTGGAATCAC 1139

CBDAS-truncated#1 TGGAATAATGGATGAGATTTCAGAATCAGCAATTCCATTCCCTCATCGAGCTGGAATCAT 131

CBDAS-truncated#2 TGGTATAATGGATGAGATTTCAGAATCAGCAATTCCATTCCCTCATCGAGCTGGAATCAT 797

CBDAS-truncated#3 TTGTCTAATGGATGAGATTTCAGAATCAGCAATTCCATTCCCTCATCGAGTTGGAATCAT 656

CBDAS-truncated#4 TGGTATAATGGATGAGATTTCAGAATCAGCAATTCCATTACCTCATTGA----------- 773

Consensus GTATGAAATTTGGTACATAGCTTCATGGGAGAAGCAAGAAGATAATGAAAAGCATATAAA 1199

THCAS GTATGAACTTTGGTACACTGCTTCCTGGGAGAAGCAAGAAGATAATGAAAAGCATATAAA 1199

CBCAS#1 GTATGAACTTTGGTACACTGCTACCTGGGAGAAGCAAGAAGATAACGAAAAGCATATAAA 1199

CBCAS#2 GTATGAACTTTGGTACACTGCTACCTGGGAGAAGCAAGAAGATAACGAAAAGCATATAAA 1199

CBCAS-truncated ------------------------------------------------------------ 1199

CBDAS#1 GTATGAAATTTGGTACATAGCTTCATGGGAGAAGCAAGAAGATAATGAAAAGCATATAAA 1025

CBDAS#2 GTATGAAATTTGGTACATAGCTTCATGGGAGAAGCAAGAAGATAATGAAAAGCATATAAA 1025

CBDAS-like#1 GTATGAAATTTGGTACATAGCTTCATGGGAGAAGCAAGAAGATAATGAAAAGCATATAAA 1061

CBDAS-like#2 GTATGAAATTTGGTACATAGCTTCATGGGAGAAGCAAGAAGATAATGAAAAGCATATAAA 1199

CBDAS-like#3 GTATGAAATTTGGTACATAGCTTCATGGGAGAAGCAAGAAGATAATGAAAAGCATATAAA 1199

CBDAS-truncated#1 GTATGAAATTTGGTACATAGCTTCATGGGAGAAGCAAGAAGATAATGAAAAGCATATAAA 191

CBDAS-truncated#2 GTATGAAATTTGGTACATAGCTTCATGGGAGAAGCAAGAAGATAATGAAAAGCATATAAA 857

CBDAS-truncated#3 GTATGAATTATGGTACATATGTAGCTGGGAGAAGCACGAAGATAAAGAAAAGTATCTAAA 716

CBDAS-truncated#4 ------------------------------------------------------------ 833

Consensus CTGGATTCGGAATGTTTATAATTTCACGACTCCTTATGTGTCCCAAAATCCAAGAATGGC 1259

THCAS CTGGGTTCGAAGTGTTTATAATTTTACGACTCCTTATGTGTCCCAAAATCCAAGATTGGC 1259

CBCAS#1 CTGGGTTCGAAGTGTTTATAATTTCACAACTCCTTATGTGTCCCAAAATCCAAGATTGGC 1259

CBCAS#2 CTGGGTTCGAAGTGTTTATAATTTCACAACTCCTTATGTGTCCCAAAATCCAAGATTGGC 1259

CBCAS-truncated ------------------------------------------------------------ 1259

CBDAS#1 CTGGATTCGGAATGTTTATAATTTCACGACTCCTTATGTGTCCCAAAATCCAAGAATGGC 1085

CBDAS#2 CTGGATTCGGAATGTTTATAATTTCACGACTCCTTATGTGTCCCAAAATCCAAGAATGGC 1085

CBDAS-like#1 CTGGATTCGGAATGTTTATAATTTCACGACTCCTTATGTGTCCCAAAATCCAAGAATGGC 1121

CBDAS-like#2 CTGGATTCGGAATGTTTATAATTTCACGACTCCTTATGTGTCCCAAAATCCAAGAATGGC 1259

CBDAS-like#3 CTGGATTCGGAATGTTTATAATTTCACGACTCCTTATGTGTCCCAAAATCCAAGAATGGC 1259

CBDAS-truncated#1 CTGGATTCAGAATGTTTACAATTTCACGACTCCTTATGTGTCCCAAAATCCAAGAATGGC 251

CBDAS-truncated#2 CTGGATTCGGAATGTTTATAATTTCACGACTCCTTATGTGTCCCAAAATCCAAGAATGGC 917

CBDAS-truncated#3 CTGGATTCGAAATGTTGATAACTTCATGACTCCTTATGTGTCCCAAAATCCAAGATTGAC 776

CBDAS-truncated#4 ------------------------------------------------------------ 893

Consensus GTATCTCAATTATAGGGACCTTGATTTAGGAAAAACTAATTTCGAGAGTCCTAATAATTA 1319

THCAS GTATCTCAATTATAGGGACCTTGATTTAGGAAAAACTAATCATGCGAGTCCTAATAATTA 1319

CBCAS#1 GTATCTCAATTATAGGGACCTTGATTTAGGAAAAACTAATCCTGAGAGTCCTAATAATTA 1319

CBCAS#2 GTATCTCAATTATAGGGACCTTGATTTAGGAAAAACTAATCCTGAGAGTCCTAATAATTA 1319

CBCAS-truncated ------------------------------------------------------------ 1319

CBDAS#1 GTATCTCAATTATAGGGACCTTGATTTAGGAAAAACTAATTTCGAGAGTCCTAATAATTA 1145

CBDAS#2 GTATCTCAATTATAGGGACCTTGATTTAGGAAAAACTAATTTCGAGAGTCCTAATAATTA 1145

CBDAS-like#1 GTATCTCAATTATAGGGACCTTGATTTAGGAAAAACTAATTTTGAGAGACCTAATAATTA 1181

CBDAS-like#2 GTATCTCAATTATAGGGACCTTGATTTAGGAAAAACTAATTTCGAGAGTCCTAATAATTA 1319

CBDAS-like#3 GTATCTCAATTATAGGGACCTTGATTTAGGAAAAACTAATTTCGAGAGTCCTAATAATTA 1319

CBDAS-truncated#1 GTATCTCAATTATAGGGACCTTGATTTAGGAAAAACTAATTTCGAGAGTCCTAATAATTA 311

CBDAS-truncated#2 GTATCTCAATTATAGGGACCTTGATTTAGGAAAAACTAATTTCGAGAGTCCTAATAATTA 977

CBDAS-truncated#3 ATATCTCAATTATAGACATCTTGATATAGGAATAAATGATCCCAAGAGTCAAAATAATTA 836

CBDAS-truncated#4 ------------------------------------------------------------ 953

Consensus CACACAAGCACGTATTTGGGGTGAAAAGTATTTTGGTAAAAATTTTAATAGGTTAGTAAA 1379

THCAS CACACAAGCACGTATTTGGGGTGAAAAGTATTTTGGTAAAAATTTTAACAGGTTAGTTAA 1379

CBCAS#1 CACACAAGCACGTATTTGGGGTGAAAAGTATTTTGGTAAAAATTTTAACAGGTTAGTTAA 1379

CBCAS#2 CACACAAGCACGTATTTGGGGTGAAAAGTATTTTGGTAAAAATTTTAACAGGTTAGTTAA 1379

CBCAS-truncated ------------------------------------------------------------ 1379

CBDAS#1 CACACAAGCACGTATTTGGGGTGAAAAGTATTTTGGTAAAAATTTTAATAGGTTAGTAAA 1205

CBDAS#2 CACACAAGCACGTATTTGGGGTGAAAAGTATTTTGGTAAAAATTTTAATAGGTTAGTAAA 1205

CBDAS-like#1 CACACAAGCACGTATTTGGGGTGAAAAGTATTTTGGTAAAAATTTTAATAGGTTAGTAAA 1241

CBDAS-like#2 CACACAAGCACGTATTTGGGGTGAAAAGTATTTTGGTAAAAATTTTAATAGGTTAGTAAA 1379

CBDAS-like#3 CACACAAGCACGTATTTGGGGTGAAAAGTAA----------------------------- 1379

CBDAS-truncated#1 CACACAAGCACGTATTTGGGGTGAAAAGTATTTTGGTAAAAAATTTAATAGGTTAGTAAA 371

CBDAS-truncated#2 CACACAAGCACGTATTTGGGGTGAAAAGTATTTTGGTAAAAATTTTAGTAGGTTAGTAAA 1037

CBDAS-truncated#3 CACAGAAGCATGTATTTTGGGTGAGAAATC-TTTGGTAAAAATTTTGACAGGCTAG---- 895

CBDAS-truncated#4 ------------------------------------------------------------ 1013

Consensus AGTAAAAACCAAGGTTGATCCCRATAATTTYTTTAGAAACGAACAAAGYATCCCACCTCT 1439

THCAS GGTGAAAACTAAAGTTGATCCCAATAATTTTTTTAGAAACGAACAAAGTATCCCACCTCT 1439

CBCAS#1 G----------------------------------------------------------- 1439

CBCAS#2 G----------------------------------------------------------- 1439

CBCAS-truncated ------------------------------------------------------------ 1439

CBDAS#1 AGTAAAAACCAAG----------------------------------------------- 1265

CBDAS#2 AGTAAAAACCAAG----------------------------------------------- 1265

CBDAS-like#1 AGTAAAAACCAAG----------------------------------------------- 1301

CBDAS-like#2 AGTAAAAACCAAG----------------------------------------------- 1439

CBDAS-like#3 ------------------------------------------------------------ 1439

CBDAS-truncated#1 AGTAAAAACCAAG----------------------------------------------- 431

CBDAS-truncated#2 AGTAAAAACCAAGGTTGATCCCGATAATTTCTTTAGAAACGAACAAAGCATCCCACCTCT 1097

CBDAS-truncated#3 ------------------------------------------------------------ 955

CBDAS-truncated#4 ------------------------------------------------------------ 1073

Consensus TCCMCYGCRTCATCATTAA 1458

THCAS TCCACCGCATCATCATTAA 1458

CBCAS#1 ------------------- 1380

CBCAS#2 ------------------- 1380

CBCAS-truncated ------------------- 969

CBDAS#1 ------------------- 1218

CBDAS#2 ------------------- 1218

CBDAS-like#1 ------------------- 1254

CBDAS-like#2 ------------------- 1392

CBDAS-like#3 ------------------- 1350

CBDAS-truncated#1 ------------------- 384

CBDAS-truncated#2 TCCCCTGCGTCATCATTAA 1116

CBDAS-truncated#3 ------------------- 891

CBDAS-truncated#4 ------------------- 762
